# Pathway engineering of *Escherichia coli* for one-step fermentative production of L-theanine from sugars and ethylamine

**DOI:** 10.1016/j.mec.2020.e00151

**Published:** 2020-11-06

**Authors:** Xiaoguang Fan, Tong Zhang, Yuanqing Ji, Jie Li, Keyi Long, Yue Yuan, Yanjun Li, Qingyang Xu, Ning Chen, Xixian Xie

**Affiliations:** a Key Laboratory of Industrial Fermentation Microbiology, Ministry of Education, Tianjin University of Science & Technology, Tianjin, 300457, PR China; b College of Biotechnology, Tianjin University of Science & Technology, Tianjin, 300457, PR China

**Keywords:** L-theanine, *Escherichia coli*, Fermentative production, Pathway engineering, γ-Glutamylmethylamide synthetase

## Abstract

L-theanine is the most abundant free amino acid in tea that offers various favorable physiological and pharmacological effects. Bacterial enzyme of γ-glutamylmethylamide synthetase (GMAS) can catalyze the synthesis of theanine from glutamate, ethylamine and ATP, but the manufacturing cost is uncompetitive due to the expensive substrates and complex processes. In this study, we described pathway engineering of wild-type *Escherichia coli* for one-step fermentative production of theanine from sugars and ethylamine. First, the synthetic pathway of theanine was conducted by heterologous introduction of a novel GMAS from *Paracoccus aminovorans*. A xylose-induced T7 RNA polymerase-P_*T7*_ promoter system was used to enhance and control *gmas* gene expression. Next, the precursor glutamate pool was increased by overexpression of native citrate synthase and introduction of glutamate dehydrogenase from *Corynebacterium glutamicum*. Then, in order to push more carbon flux towards theanine synthesis, the tricarboxylic acid cycle was interrupted and pyruvate carboxylase from *C. glutamicum* was introduced as a bypath supplying oxaloacetate from pyruvate. Finally, an energy-conserving phosphoenolpyruvate carboxykinase from *Mannheimia succiniciproducens* was introduced to increase ATP yield for theanine synthesis. After optimizing the addition time and concentration of ethylamine hydrochloride in the fed-batch fermentation, the recombinant strain TH11 produced 70.6 ​g/L theanine in a 5-L bioreactor with a yield and productivity of 0.42 ​g/g glucose and 2.72 ​g/L/h, respectively. To our knowledge, this is the first report regarding the pathway engineering of *E. coli* for fermentative production of theanine. The high production capacity of recombinant strain, combined with the easy processes, will hold attractive industrial application potential for the future.

## Highlights

• γ-Glutamylmethylamide synthetase from *P. aminovorans* showed high ligation activity.
• Xylose-induced T7 RNA polymerase-P_*T7*_ promoter system was used to control gene expression.
• TCA cycle was rewired to push more carbon flux toward theanine synthesis.
• Ethylamine feeding strategy was optimized to balance cell growth and theanine production.

## 1 Introduction

Tea is the most consumed hot drink and the most popular non-alcoholic beverage worldwide. The unique taste and health benefits of tea are associated with the abundant secondary metabolites in tea plants (*Camellia sinensis*), including polyphenols, alkaloids, volatile oils and amino acids (Li et al., 2017). L-theanine (further referred to as theanine) is a main bioactive component in *C. sinensis* and accounts for approximately 50% of the free amino acids (Li et al., 2017). Theanine was certified as generally regarded as safe (GRAS) ingredient by USFDA, and has been widely used in food and pharmaceutical industries (Türközü and Şanlier, 2017). Aside from its flavor-enhancing ability, theanine is reported to have favorable physiological and pharmacological effects, such as reducing chronic stress response and anxiety levels (Lu et al., 2004; Hidese et al., 2019), improving sleep quality and cognitive function (Hidese et al., 2019; Kim et al., 2019), and alleviating some symptoms of schizophrenia, cancer and cardiovascular diseases (Türközü and Şanlier, 2017; Sakamoto et al., 2019).

Theanine can be extracted from tea leaves by the use of resins or preparative HPLC, but the yield and purity are low since the content of theanine in tea leaves is only about 7–21 ​mg/g of dry weight (Zhang et al., 2004; Vuong et al., 2011). Chemical synthesis provides simple and cost effective approach for high-purity theanine production, but the synthetic products are racemic mixture that are difficult to separate (Vuong et al., 2011). Biological production of theanine has many advantages over other production techniques, such as stereo-selectiveness, high specificity, simple reaction, mild condition and little chemical reagents. In *C. sinensis*, theanine is synthesized by addition of ethylamine to glutamate in an ATP-dependent manner catalyzed by theanine synthetase (TS, EC 6.3.1.6) (Deng et al., 2008). Because of its low stability, the plant-derived TS cannot be used to produce theanine in commercial quantities. Therefore, bacterial enzymes such as L-glutaminase (EC 3.5.1.2) (Pu et al., 2013), *γ*-glutamyltranspeptidase (GGT, EC 2.3.2.2) (Bindal and Gupta, 2014), L-glutamine synthetase (GS, EC 6.3.1.2) (Yamamoto et al., 2006), *γ*-glutamylmethylamide synthase (GMAS, EC 6.3.4.12) (Yamamoto et al., 2008a), and *γ*-glutamylcysteine synthetase (γ-GCS, EC 6.3.2.2) (Miyake and Kakita, 2009) have been developed for theanine synthesis. L-glutaminase and GGT can directly transfer a glutamyl moiety to ethylamine without ATP requirement, but their substrate glutamine is easy to be hydrolyzed to glutamate or be transformed to *γ*-glutamylglutamine during whole-cell catalysis process (Mu et al., 2019). GS, GMAS, and γ-GCS catalyze similar reactions like TS, but their activities toward glutamate and ethylamine are different. Among them, GMAS showed much higher ligation activity of γ-glutamyl group towards ethylamine. GMAS derived from *Methylovorus mays* No. 9 showed 100-fold activity for ethylamine than GS from *Pseudomonas taetrolens* Y-30, producing approximately 110 ​g/L theanine with the ATP regeneration system of yeast sugar fermentation, which represents the highest theanine production ever reported (Yamamoto et al., 2008a, 2008b). Recently, semi-rational mutagenesis was used to switch the specificity of substrate binding pockets of γ-GCS from *Escherichia coli* (Yao et al., 2020). The mutant enzyme 13B6 showed 14.6% decrease in the specific activity for the original substrate cysteine, but exhibited 14.6- and 17.0-fold improvements in theanine production and catalytic efficiency for ethylamine, respectively.

Biotransformation of theanine using immobilized enzymes or whole cell catalysts had industrial application potential, but all approaches required external supply of expensive substrates including glutamine, glutamate or even ATP. Although ATP could be recycled by the use of polyphosphate kinase (PPK) (Liu et al., 2016) or provided by yeast sugar fermentation (Yamamoto et al., 2008b), the introduction of the ATP regeneration systems further increased the reaction cost and operation complexity. Fermentative production of theanine and N-methylglutamate can be realized based on a pathway for methylamine assimilation found in some methylotrophs, such as *Methylobacterium extorquens*, *Methylocella silvestris*, and *Methyloversatilis universalis* (Gruffaz et al., 2014; Chen et al., 2010; Latypova et al., 2010). In our recent study, plasmid-based overexpression of *gmas* gene from *M. mays* No. 9 (*gmas*_*Mm*_) in a glutamate overproducing strain *Corynebacterium glutamicum* GDK-9 enabled a glucose and ethylamine dependent fermentative production of theanine (Ma et al., 2020). The prevention of glutamate export by deletion of the exporter gene *cgl1221* improved theanine titer to 42 ​g/L with a yield of 19.6% in a 5-L bioreactor. However, chromosome-based overexpression of *gmas*_*Mm*_ in GDK-9 led to a significant decrease in theanine production. This is a common challenge faced when expressing key genes in the metabolic engineering of *C. glutamicum* (Ma et al., 2020; Zhang et al., 2018). Moreover, wild-type strain *C. glutamicum* ATCC 13032 harboring the designed plasmid produced considerable less theanine, indicating that systematic integration of constructed pathways with host metabolism is crucial for the construction of genetically defined theanine producers with higher performance.

**Fig. 1:**
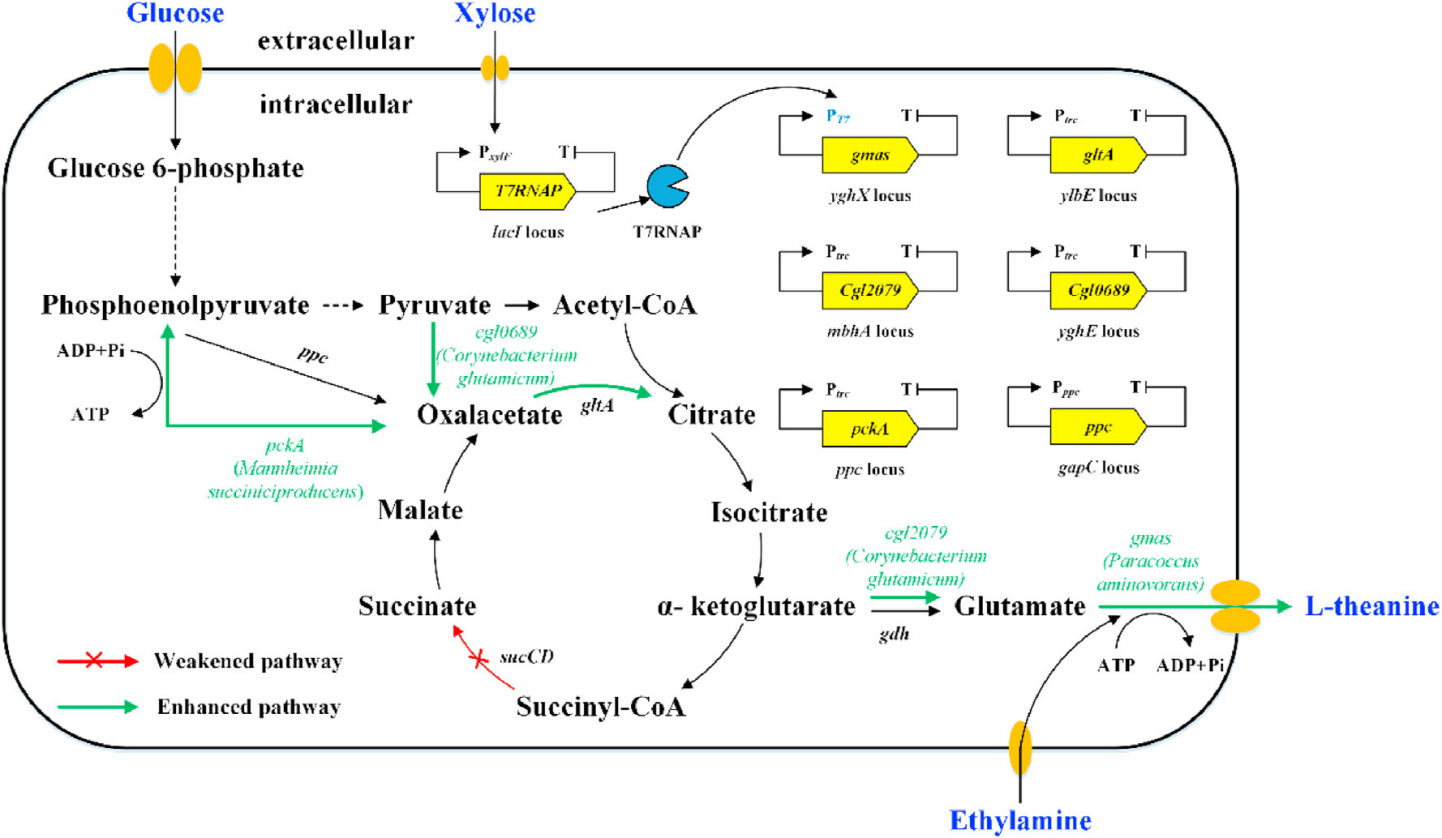
Overall metabolic engineering strategies for theanine overproduction in E.coli. ppc: pyruvate carboxylase gene; gltA: citrate synthase gene; sucCD: succinyl-CoA synthetase gene; pckA: phosphoenolpyruvate carboxykinase gene from M. succiniciproducens; cgl0689: pyruvate carboxylase gene from C. glutamicum GDK-9; cgl2079: glutamate dehydrogenase gene from C. glutamicum GDK-9; gmas: *γ*-glutamylmethylamide synthase gene from P. aminovorans.

Compared with *C. glutamicum*, *E. coli* is an easier-to-engineer host for production of numbers of amino acids (Niu et al., 2019, Wendisch, V. F. 2020). It processes a well-characterized genetic background and grows rapidly in cheap media. Moreover, numerous molecular tools have been developed for enhancing gene expression in *E. coli* at the transcriptional and translational levels (Kondo and Yumura, 2020; Nakashima et al., 2014). Hence, we attempted to obtain genetically defined theanine producers in this study by systematically engineering the synthetic pathway in *E. coli* (Fig. 1). A novel GMAS from *Paracoccus aminovorans* (GMAS_*Pa*_) was selected and characterized, which showed higher specific activity than that from other methylotrophs. A xylose-induced T7 RNA polymerase-P_*T7*_ promoter system was used to optimize the expression level of GMAS_*Pa*_. Rational strategies were applied to redirect metabolic flux from the tricarboxylic acid (TCA) cycle towards the constructed theanine biosynthetic pathway, including enrichment of glutamate synthesis, interruption of succinate synthesis, introduction of pyruvate carboxylation pathway, and reinforcement of ATP generation. The feeding strategies of ethylamine in fed-batch fermentation process were optimized to balance cell growth and theanine production. The final strain TH11 produced 70.6 ​g/L theanine in a 5-L bioreactor with a yield and productivity of 0.42 ​g/g glucose and 2.72 ​g/L/h, respectively. To our knowledge, this is the first report regarding the pathway engineering of *E. coli* for fermentative production of theanine.

## 2 Materials and methods

### 2.1 Strains and plasmids

The strains and plasmids used in this study are listed in Table 1. *E. coli* DH5α and *E. coli* BL21(DE3) were used as host for cloning and gene expression, respectively. *E. coli* W3110 was used as the starting strain for genomic manipulations. The plasmids pREDCas9 and pGRB used for CRISPR/Cas9-mediated gene editing system were kindly provided by Prof. Tao Chen of Tianjin University (Li et al., 2015). The plasmid pET28a was used to construct expression vectors (Ma et al., 2020).

**Table 1:** Strains and plasmids used in this study.

Strains/plasmids	Characteristics	Source
Strains		
*E. coli* DH5α	Host for cloning	Lab stock
*E. coli* BL21(DE3)	Host for gene expression	Lab stock
*E. coli* W3110	Wild type, starting strain	Lab stock
TH1	W3110, △*lacI*, *yghX*::P_*trc*_*-gmas*_*Mu*_ (*Methyloversatilis universalis*)	This study
TH2	W3110, △*lacI*, *yghX*::P_*trc*_*-gmas*_*Me*_ (*Methyloversatilis extorquens*)	This study
TH3	W3110, △*lacI*, *yghX*::P_*trc*_*-gmas*_*Mm*_ (*Methylovorus mays*)	This study
TH4-1	W3110, △*lacI*, *yghX*::P_*trc*_*-gmas*_*Pa*_ (*Paracoccus aminovorans*)	This study
TH4-2	TH4-1, *yeeP*::P_*trc*_*-gmas*_*Pa*_	This study
TH4-3	TH4-2, *mbhA*::P_*trc*_*-gmas*_*Pa*_	This study
TH5-1	W3110, *lacI*::P_*xylF*_*-T7RNAP*, *mlc*::*mlc∗*, *yghX*::P_*T7*_*-gmas*_*Pa*_	This study
TH5-2	TH5-1, *yeeP*::P_*T7*_*-gmas*_*Pa*_	This study
TH6	TH5-1, *ylbE*::P_*trc*_*-gltA*	This study
TH7	TH6, *mbhA*::P_*trc*_*-cgl2079* (*Corynebacterium glutamicum*)	This study
TH8	TH7, △*sucCD*	This study
TH9	TH8, *yghE*::P_*trc*_*-cgl0689* (*Corynebacterium glutamicum*)	This study
TH10	TH9, *ppc*::P_*trc*_*-pckA*_*Ms*_ (*Mannheimia succiniciproducens*)	This study
TH11	TH10, *gapC*::*ppc*	This study
**Plasmids**		
pREDCas9	Spe^r^, Cas9 and λ Red recombinase expression vector	Li et al. (2015)
pGRB	Amp^r^, gRNA expression vector	Li et al. (2015)
pET28a	f1 origin, *lacI*, *T7* promoter, Kan^r^, expression vector	Lab stock
pET28a-*gmas*_*mu*_	Plasmid for *gmas*_*mu*_ expression	This study
pET28a-*gmas*_*me*_	Plasmid for *gmas*_*me*_ expression	This study
pET28a-*gmas*_*mm*_	Plasmid for *gmas*_*mm*_ expression	This study
pET28a-*gmas*_*pa*_	Plasmid for *gmas*_*pa*_ expression	This study

### 2.2 GMAS expression, purification and enzyme activity assays

GMAS genes from *M. universalis* (WP_018227792.1), *M. extorquens* (WP_015822233.1), *M. mays* (BAF99006.1), and *P. aminovorans* (SFH87749.1) were codon-optimized and synthesized by Genewiz (Suzhou, China). These gene fragments were individually ligated into the plasmid pET-28a using the EcoR I/Hind III restriction sites, and the recombinant plasmids were individually transformed into *E. coli* BL21(DE3) competent cells by electroporation. The transformants were cultured in LB medium with 50 ​μg/mL kanamycin at 37 ​°C. When the cell density (OD_600_) reached 0.6~0.8, 0.1 ​mM isopropyl β-D-1-thiogalactopyranoside (IPTG) was added to induce protein expression at 18 ​°C for 12 ​h. The harvested cells were resuspended in buffer (50 ​mM Na_2_HPO_4_ and 300 ​mM NaCl) and disrupted by a high-pressure cell crusher (UH-03, Union-Biotech, Shanghai, China). Cell debris was removed by centrifugation and the His_6_-tag enzymes in the supernatants were trapped on Ni-NTA superflow resin (Qiagen, Hilden, Germany). After washing with buffer (20 ​mM Tris-HCl (pH 8.0), 300 ​mM imidazole, 0.5 ​M NaCl and 1 ​mM dithiothreitol (DTT), the eluant containing purified enzymes was dialyzed and collected.

GMAS activity was assayed according to our previous report (Ma et al., 2020). One unit of enzyme activity was defined as the amount of enzyme that catalyzes the formation of 1 ​μmol of theanine per minute. The protein concentration was measured using a BCA protein assay kit (Solarbio, Beijing, China).

### 2.3 Strain construction via CRISPR/Cas9-mediated gene editing system

The chromosomal integration or deletion of genes in *E. coli* W3110 was performed via CRISPR/Cas9-mediated gene editing system. All the primers used for strain construction are listed in Table S1. The glutamate dehydrogenase (GDH) gene (WP_003856385.1) and pyruvate carboxylase (PYC) gene (WP_011013816.1) from *C. glutamicum* were directly amplified by PCR. The phosphoenolpyruvate carboxykinase (PCK) gene (WP_011201442.1) from *Mannheimia succiniciproducens* was codon-optimized and synthesized by Genewiz. To construct the donor DNA fragments for integration, the up- and down-stream homology arms and the target sequence were amplified and fused together by the overlapping PCR. To construct the gRNA plasmid, the 20 bp spacer specific for each target was designed using CRISPR RGEN Tools (http://www.rgenome.net/) and ligated into the plasmid pGRB using a ClonExpress II one step cloning kit (Vazyme, Nanjing, China).

The plasmid pREDCas9 was first transformed into *E. coli W3110* competent cells. The transformants were cultured in LB medium with 50 ​μg/mL spectinomycin at 37 ​°C. When the OD_600_ reached 0.1~0.2, 0.1 ​mM IPTG was added to induce the expression of λ red recombinase. When the OD_600_ reached 0.6~0.7, appropriate amounts of the donor DNA fragments and the gRNA plasmid were co-transformed into the competent cells, and the transformants were cultured in LB medium with 50 ​μg/mL spectinomycin and 50 ​μg/mL ampicillin at 37 ​°C. The individual colonies were identified by colony PCR, and the randomly selected positive transformants were confirmed by DNA sequencing. The gRNA plasmid can be eliminated by culturing the correct colonies in LB medium with 0.2% L-arabinose (Li et al., 2015). The plasmid pREDCas9 can be eliminated by raising the culture temperature from 37 ​°C to 42 ​°C (Li et al., 2015).

### 2.4 Shake-flask fermentation

A loop of engineered strains grown on an agar slant were transferred to 30 ​mL seed-culture medium in a 500 ​mL shake flask and cultivated at 37 ​°C with a shaking rate of 200 ​rpm for 10 ​h. The seed-culture medium contained (per liter) 25 ​g glucose, 5 ​g yeast extract, 3 ​g tryptone, 1.2 ​g KH_2_PO_4_, and 0.5 ​g MgSO_4_·7H_2_O, with initial pH adjusted to 7.0~7.2 by NaOH. The seed cultures (3 ​mL) were then transferred to 30 ​mL fermentation medium in a 500 ​mL baffled shake flask and cultivated at 37 ​°C with a shaking rate of 200 ​rpm for 24 ​h. The fermentation medium contained (per liter) 20 ​g glucose, 4 ​g yeast extract, 3 ​g tryptone, 2 ​g sodium citrate, 2 ​g KH_2_PO_4_, 2 ​g MgSO_4_·7H_2_O, 10 ​mg FeSO_4_·7H_2_O, and 10 ​mg MnSO_4_·H_2_O, with initial pH adjusted to 7.0~7.2 by NaOH. Ammonium hydroxide (25%, v/v) was added with a microinjector to maintain the pH at approximately 7.0, with phenol red as the pH indicator. During the fermentation, the pH value was controlled by addition of ammonium hydroxide (25%, v/v) based on the color change of phenol red. At 9 ​h of the fermentation, xylose was one-time added with a final concentration of 5 ​g/L to induce the expression of GMAS. At the same time, 5 ​mL of ethylamine hydrochloride (16%, m/v) was supplemented at 3 ​h intervals. When necessary, glucose (60%, m/v) was added with a microinjector to satisfy the glucose demand for cell growth and theanine production.

### 2.5 Fed-batch fermentation in a 5-L bioreactor

An appropriate amount of agar slant cultured cells were transferred to 2 ​L seed medium in a 5-L bioreactor (Baoxing, Shanghai, China). The seed medium in the bioreactor was the same as that used in the shake flask. The pH was automatically controlled by the addition of ammonium hydroxide (25%, v/v) and the temperature was set at 37 ​°C. Dissolved oxygen was maintained above 20% by variation of the stirrer speed and the aeration rate. When the OD_600_ reached 12~15, a certain seed culture was kept (250 ​mL), and the fresh fermentation medium (2.5 ​L) was immediately supplemented. The fermentation medium contained (per liter) 20 ​g glucose, 4 ​g yeast extract, 3 ​g tryptone, 2 ​g sodium citrate, 2 ​g KH_2_PO_4_, 2 ​g MgSO_4_·7H_2_O, 10 ​mg FeSO_4_·7H_2_O, 10 ​mg MnSO_4_·H_2_O, 1 ​mg V_B1_, 1 ​mg V_B3_, 1 ​mg V_B5_, 1 ​mg V_B12_, and 1 ​mg V_H_, with initial pH adjusted to 7.0~7.2 by NaOH. The pH was automatically controlled by the addition of ammonium hydroxide (25%, v/v) and the temperature was set at 37 ​°C. Dissolved oxygen was maintained above 20% by variation of the stirrer speed and the aeration rate. Xylose was one-time added with a final concentration of 5 ​g/L to induce the expression of GMAS at a certain time (4, 6 or 8 ​h). At the same time, ethylamine hydrochloride (12–24%, m/v) was automatically fed at a rate of 30 ​mL/h for 20 ​h. When necessary, glucose (80%, m/v) was fed at an appropriate rate to maintain the glucose concentration below 5 ​g/L.

### 2.6 Analytical methods

Cell growth was monitored by measuring the absorbance at 600 ​nm (OD_600_). theanine and other amino acids in the cultures was quantified according to our previous report (Ma et al., 2020; Zhang et al., 2018). Acetate, formate and glucose concentration were determined by HPLC (Shimadzu, Japan), using an Aminex HPX-87H column (Bio-Rad, Hercules, CA, USA) at 30 ​°C with a refractive index detector. As the mobile phase, 5 ​mM sulfuric acid was supplied at a flow rate of 0.5 ​mL/min.

### 2.7 Statistical analysis

All fermentation experiments were performed in triplicate. For each step of genetic manipulation, the difference between the data obtained from the engineered strain and the relevant control strain was analyzed via one-way analysis of variance (ANOVA), followed by student’s t-test using a two-tailed distribution. A value of p ​< ​0.05 was considered significant, while p ​< ​0.01 was considered highly significant.

## 3 Results

### 3.1 Selection of GMAS with high theanine forming activity

**Fig. 2:**
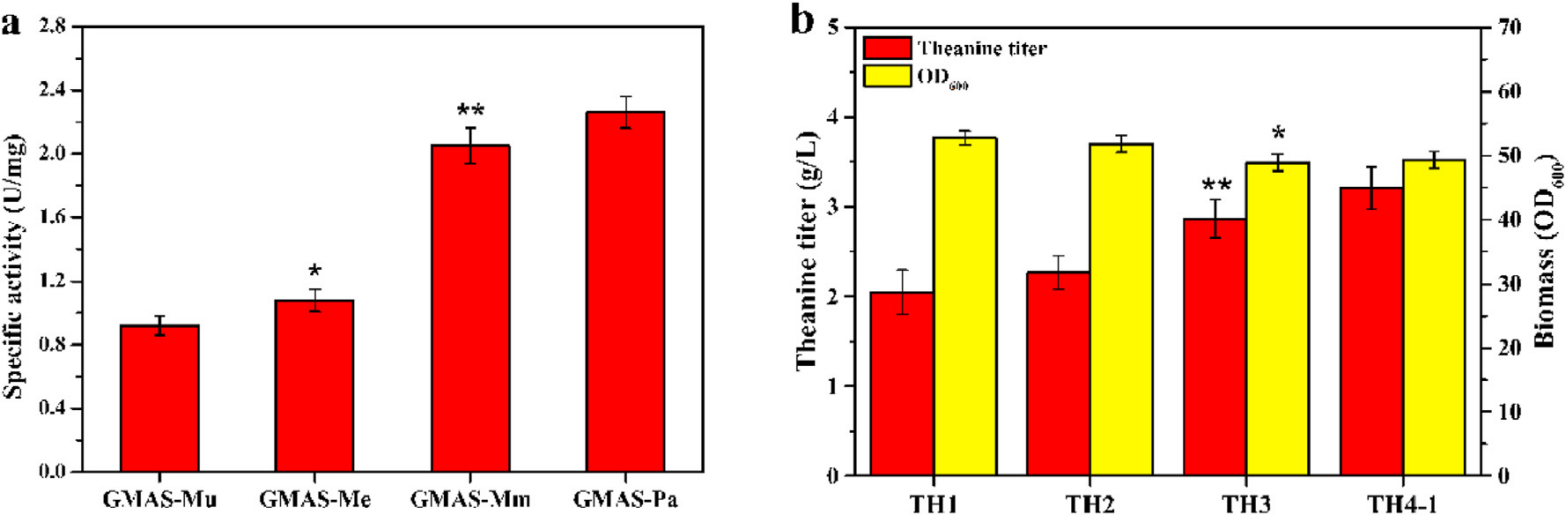
Selection of GMAS for the fermentative production of theanine. **a** Specific activities of GMAS_*Mu*_, GMAS_*Me*_, GMAS_*Mm*_ and GMAS_*Pa*_ expressed in *E. coli* BL21(DE3). **b** Effect of integrating different P_*trc*_-*gmas* gene in *E. coli* W3110 on theanine titer and biomass. Each experiment was repeated three times, and data are represented as the means of three replicates and bars represent the standard deviations. ∗, P ​< ​0.05 and ∗∗, P ​< ​0.01 indicate the significance level between the two engineered strain. e.g., GMAS-Mu v GMAS-Me, GMAS-Mm v GMAS-Me, GMAS-Pa v GMAS-Mm, TH2 v TH1, TH3 v TH2, TH4-1 v TH3.

GMAS plays an important role in the methylamine metabolism in methylotrophs and thus shows high activity towards amines. The GMAS enzymes derived from *M. universalis* (GMAS_*Mu*_), *M. extorquens* (GMAS_*Me*_), and *M. mays* (GMAS_*Mm*_) have been successfully expressed in *E. coli* BL21(DE3) and purified for the catalytic reaction of theanine in our previous study (Ma et al., 2020). In this study, we examined another methylotrophic strain, *P. aminovorans* JCM7685, which was isolated from a sample of soil contaminated with dimethylformamide and showed high degradative capabilities towards organic compounds (Urakami et al., 1990). SDS-PAGE analysis (Figure S1) revealed that GMAS_*pa*_ were expressed in both active form and inclusion bodies form in *E. coli* BL21(DE3), and its expression level was higher than other GMAS enzymes (Ma et al., 2020). Moreover, the specific activity of GMAS_*Pa*_ (Fig. 2) was almost two-fold increase over that of GMAS_*Mu*_ and GMAS_*Me*_, and 10.2% higher than that of GMAS_*Mm*_.

To further determine the capacities of the four GMAS enzymes for the fermentative production of theanine, these GMAS genes were separately placed under the P_*trc*_ promoter and integrated into the pseudogene *yghX* locus of *E. coli* W3110. The *lacI* gene was deleted to obtain the constitutive expression of genes under P_*trc*_ promoter. The resulting strains were cultivated in shake-flask fermentation medium supplemented with ethylamine. As shown in Fig. 2b, chromosome-based expression of *gmas* resulted in the accumulation of theanine in the fermentation broth, indicating that ethylamine could be taken up and theanine could be secreted by *E. coli* cells. TH4-1 (P_*trc*_-*gmas*_*Pa*_) produced the highest titer of theanine (3.2 ​g/L), which was 60.0%, 39.1%, and 10.3% higher than that produced by TH1 (P_*trc*_-*gmas*_*Mu*_), TH2 (P_*trc*_-*gmas*_*Me*_) and TH3 (P_*trc*_-*gmas*_*Mm*_), respectively. The biomass of TH4-1 was almost the same as that of TH3, but lower than that of TH1 and TH2, indicating that intracellular expression of GMAS enzyme with high expression level and high specific activity would affect the growth of *E. coli* cells to some extent.

### 3.2 Controllable synthesis of theanine by a xylose-induced T7 RNA polymerase-P_*T7*_ promoter system

**Fig. 3:**
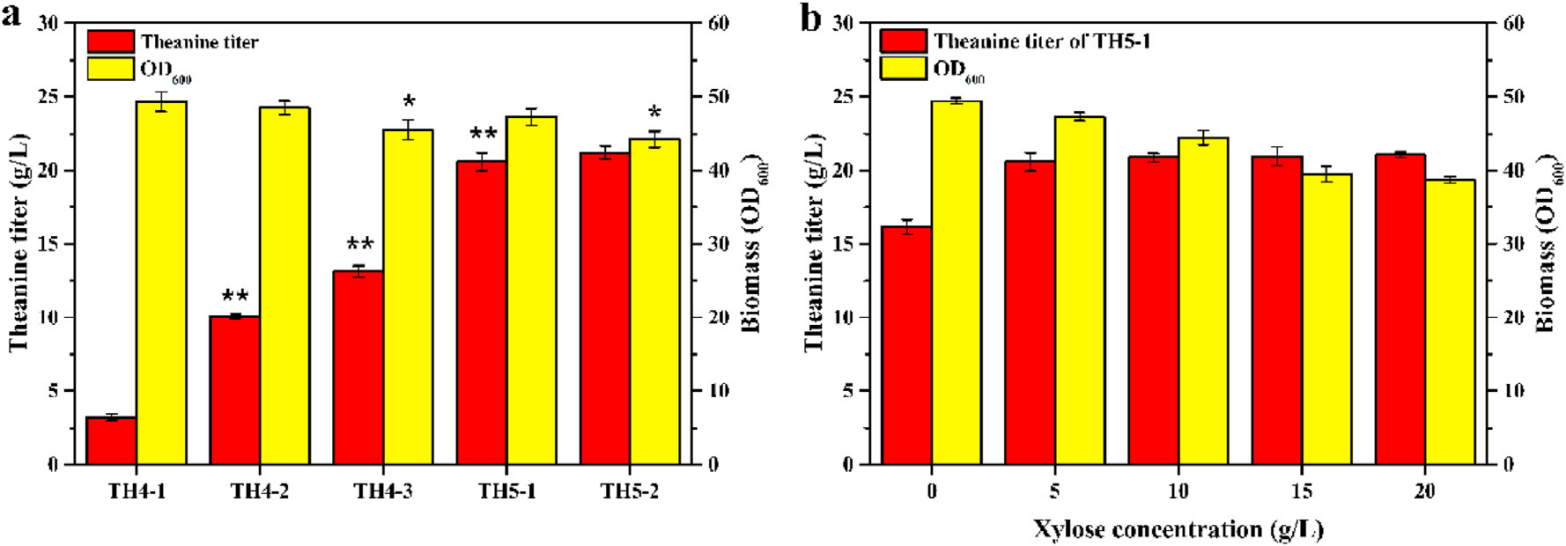
Optimization of the expression levels of *gmas*_*Pa*_ for the fermentative production of theanine. a Optimization of the promoter and copy numbers of *gmas*_*Pa*_. b Optimization of xylose inducer concentration. TH4-1: one copy of P_*trc*_-*gmas*_*Pa*_; TH4-2: two copies of P_*trc*_-*gmas*_*Pa*_; TH4-3: three copies of P_*trc*_-*gmas*_*Pa*_; TH5-1: one copy of P_*T7*_-*gmas*_*Pa*_; TH5-2: two copies of P_*T7*_-*gmas*_*Pa*_. Each experiment was repeated three times, and data are represented as the means of three replicates and bars represent the standard deviations. ∗, P ​< ​0.05 and ∗∗, P ​< ​0.01 indicate the significance level between the two engineered strain. e.g., TH4-2 v TH4-1, TH4-3 v TH4-2, TH5-1 v TH4-3, TH5-2 v TH5-1.

As *gmas* is the rate-limiting enzyme for theanine synthesis, we first enhanced the expression of *gmas*_*pa*_ by introducing more copies in TH4-1. Another one or two copies of *gmas*_*Pa*_ under the control of P_*trc*_ promoter were integrated into the pseudogene *yeep* and *mbhA* locus of TH4-1, generating TH4-2 and TH4-3, respectively. As shown in Fig. 3a, the theanine titer of TH4-2 (10.1 ​g/L) was 3.2-fold increase over that of TH4-1, while the biomass of TH4-2 was almost the same as that of TH4-1. Further raising the copy numbers of P_*trc*_-*gmas*_*Pa*_ on the chromosome resulted in a 29.7% improvement in theanine titer (13.1 ​g/L), but the biomass was decreased by 7.9%.

Considering that constitutive expression of three copies of P_*trc*_-*gmas*_*Pa*_ repressed cell growth, we then used a “xylose-induced T7 RNA polymerase-P_*T7*_ promoter system” to achieve the controllable synthesis of theanine. The T7 RNA polymerase gene under the control of P_*xylF*_ promoter was first integrated into the *lacI* locus of *E. coli* W3110, and the promoter region of *mlc* gene encoding a global transcriptional repressor was mutated to relieve the carbon catabolite repression (Nakashima and Tamura, 2012). Then, one or two copies of *gmas*_*Pa*_ under the control of P_*T7*_ promoter were integrated into the pseudogene *yeep* and *mbhA* locus, generating TH5-1 and TH5-2, respectively. Xylose was one-time added at 9 ​h of the fermentation with a final concentration of 5 ​g/L to induce the *gmas*_*Pa*_ expression. As shown in Fig. 3a, the theanine titer of TH5-1 (20.6 ​g/L) was 57.2% higher than that of TH4-3, because the expression level from P_*T7*_ was much higher compared to P_*trc*_ (Figure S2). Moreover, the biomass of TH5-1 was 4.2% higher than that of TH4-3, suggesting that expression control of P_*T7*_-*gmas*_*Pa*_ via xylose induction was a beneficial manner to balance the cell growth and product synthesis. However, TH5-2 with two copies of P_*T7*_-*gmas*_*Pa*_ showed no obvious increase in theanine production (21.2 ​g/L), whereas its biomass was reduced by 6.6%. Hence, we chose TH5-1 as target strain for follow-up study.

In order to determine the optimal xylose induction conditions, xylose with different concentration was added at 9 ​h of the fermentation. As shown in Fig. 3b, when xylose concentration ranged from 5 ​g/L to 20 ​g/L, the theanine titer did not changed significantly (from 20.6 ​g/L to 21.0 ​g/L), but the biomass (OD_600_) was decreased by 18.2%, indicating that 5 ​g/L xylose was sufficient for *gmas*_*Pa*_ expression and theanine production. It was worth noting that 16.1 ​g/L theanine was produced in the absence of xylose, indicating that baseline expression from the “xylose-induced T7 RNA polymerase-P_*T7*_ promoter system” was high (Nakashima et al., 2014).

### 3.3 Enrichment of the precursor glutamate pool

**Fig. 4:**
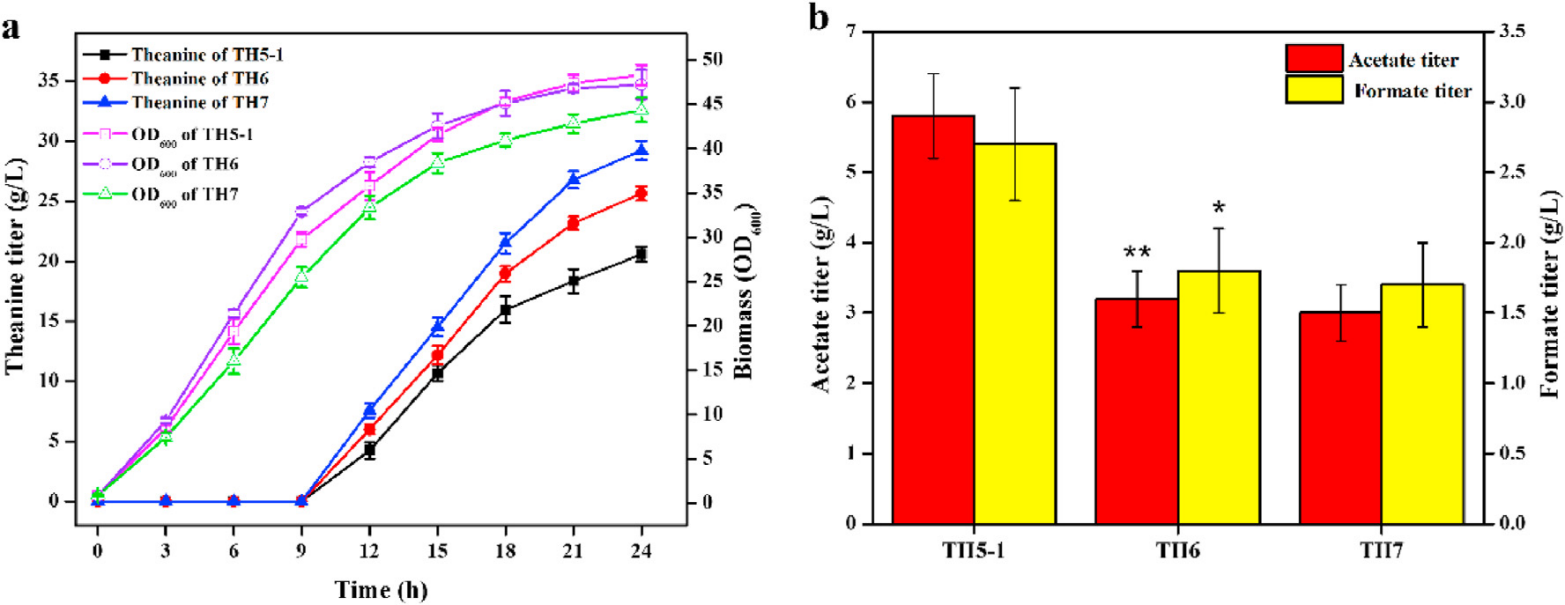
Effect of overexpressing the native citrase synthase (TH6) and glutamate dehydrogenase from *C. glutamicum* GDK-9 (TH7) on theanine titer and biomass (**a**), and the formation of acetate and formate (**b**). Each experiment was repeated three times, and data are represented as the means of three replicates and bars represent the standard deviations. ∗, P ​< ​0.05 and ∗∗, P ​< ​0.01 indicate the significance level between the two engineered strain. e.g., TH6 v TH5-1, TH7 v TH6.

Citrase synthase (CS) catalyzes the condensation reaction of oxaloacetate and acetyl-CoA, which governs the carbon flux into TCA cycle. TCA cycle can supply efficient precursors as well as ATP for theanine synthesis. Hence, we enhanced the CS activity by overexpressing the native CS gene *gltA* (Chen et al., 2020a). Another copy of *gltA* controlled by P_*trc*_ promoter was integrated into the pseudogene *ylbe* locus of TH5-1, generating strain TH6. As expected, the theanine titer of TH6 (25.6 ​g/L) was increased by 24.3% compared with that of TH5-1 (Fig. 4a). The growth rate of TH6 was obviously greater than that of TH5-1 ​at the beginning of fermentation, but the final biomass was almost the same. In addition, the acetate and formate titer of TH6 were reduced by 44.8% and 33.3% compared to that of TH5-1 (Fig. 4b), respectively, indicating that part of carbon flux from pyruvate was channeled to the TCA cycle.

Glutamate dehydrogenase (GDH) is an enzyme involved in the synthesis of glutamate by reductive amination reaction of α-ketoglutarate. Enhancing the GDH activity is proved to be an effective strategy for increasing the production of glutamate-derived compounds (Jensen et al., 2015). *C. glutamicum* is a natural glutamate producer due to the high level of GDH activity. Hence, GDH gene *cgl2079* from *C. glutamicum* GDK-9 was placed under the P_*trc*_ promoter and integrated into the pseudogene *ylbE* locus of TH6, generating strain TH7. As shown in Fig. 4a, the theanine titer of TH7 (29.2 ​g/L) was increased by 14% compared with that of TH6, while the growth rate and final biomass were slightly decreased. These results indicated that more α-ketoglutarate in TCA cycle was pulled into glutamate and theanine biosynthetic pathways, and thus led to decrease the carbon and energy for cell growth (Xu et al., 2018). In addition, introduction of heterogenous GDH did not affect the acetate and formate accumulation under aerobic conditions (Fig. 4b).

### 3.4 Rewiring the TCA cycle for theanine production

**Fig. 5:**
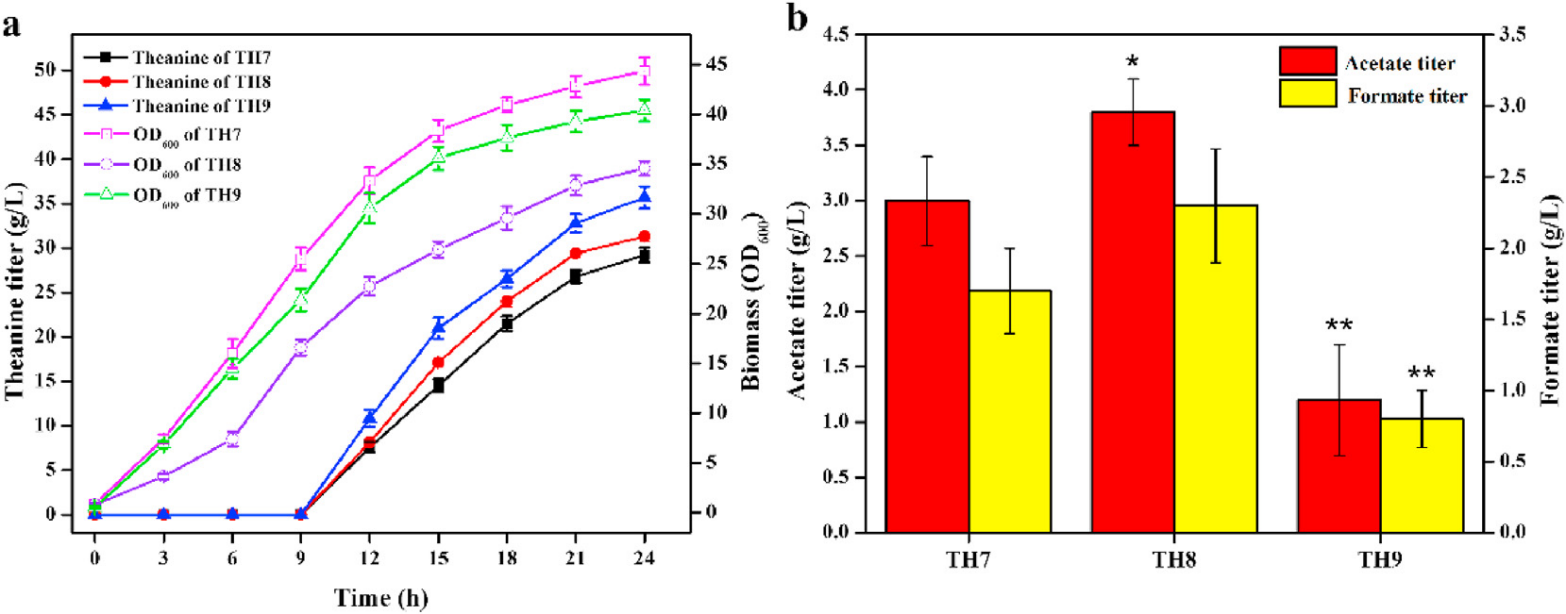
Effect of knocking out succinyl-CoA synthetase (TH8) and overexpressing pyruvate carboxylase from *C. glutamicum* GDK-9 (TH9) on theanine titer and biomass (**a**), and the formation of acetate and formate (**b**). Each experiment was repeated three times, and data are represented as the means of three replicates and bars represent the standard deviations. ∗, P ​< ​0.05 and ∗∗, P ​< ​0.01 indicate the significance level between the two engineered strain. e.g., TH8 v TH7, TH9 v TH8.

Because α-ketoglutarate is an intermediate in the TCA cycle, the next modification aimed at reducing its degradation and saving more carbon flux for theanine production. Hence, we attempted to interrupt the metabolism of succinyl-CoA to succinate by knocking out the succinyl-CoA synthetase gene *sucCD* in TH7, generating strain TH8. As a result, TH8 showed obvious growth inhibition, whereas its theanine titer (30.3 ​g/L) was comparable with that of TH7 (Fig. 5a). In addition, the acetate and formate titer of TH8 were increased by 26.7% and 35.3% compared to that of TH7 (Fig. 5b), respectively. These results indicated that though the metabolic flux for cell growth was decreased, the excess flux was not redirected to theanine production. This might be due to lack of oxaloacetate, which is the precursor of entrance reaction of TCA cycle, resulting from the interruption of succinate synthesis (Soma et al., 2017).

Anaplerotic pathways can replenish oxaloacetate by carboxylation of either phosphoenolpyruvate or pyruvate. In contrast to *E. coli*, *C. glutamicum* possesses two anaplerotic enzymes, i.e., phosphoenolpyruvate carboxylase (PPC) and pyruvate carboxylase (PYC) (Kortmann et al., 2019). Although both of them played a part in the supply of oxaloacetate in *C. glutamicum*, PYC is proved to be the major enzyme involved in fulfilling the oxaloacetate demand for the production of glutamate (Peterswendisch et al., 2001). Thus, PYC gene *cgl0689* from *C. glutamicum* GDK-9 was placed under the P_*trc*_ promoter and integrated into the pseudogene *yghE* locus of TH8, generating strain TH9. As shown in Fig. 5a, the theanine titer of TH9 (35.7 ​g/L) was improved by 17.5% compared with that of TH8, and the cell growth of TH9 was restored. However, the growth rate and the final biomass of TH9 were still lower than that of TH7, respectively. In addition, the acetate and formate titer of TH9 were significantly reduced by 68.4% and 65.2% compared to that of TH8 (Fig. 5b), respectively. These results indicated that the metabolic flux in the TCA cycle was successfully redirected to the theanine biosynthetic pathway.

### 3.5 Reinforcement of ATP generation

**Fig. 6:**
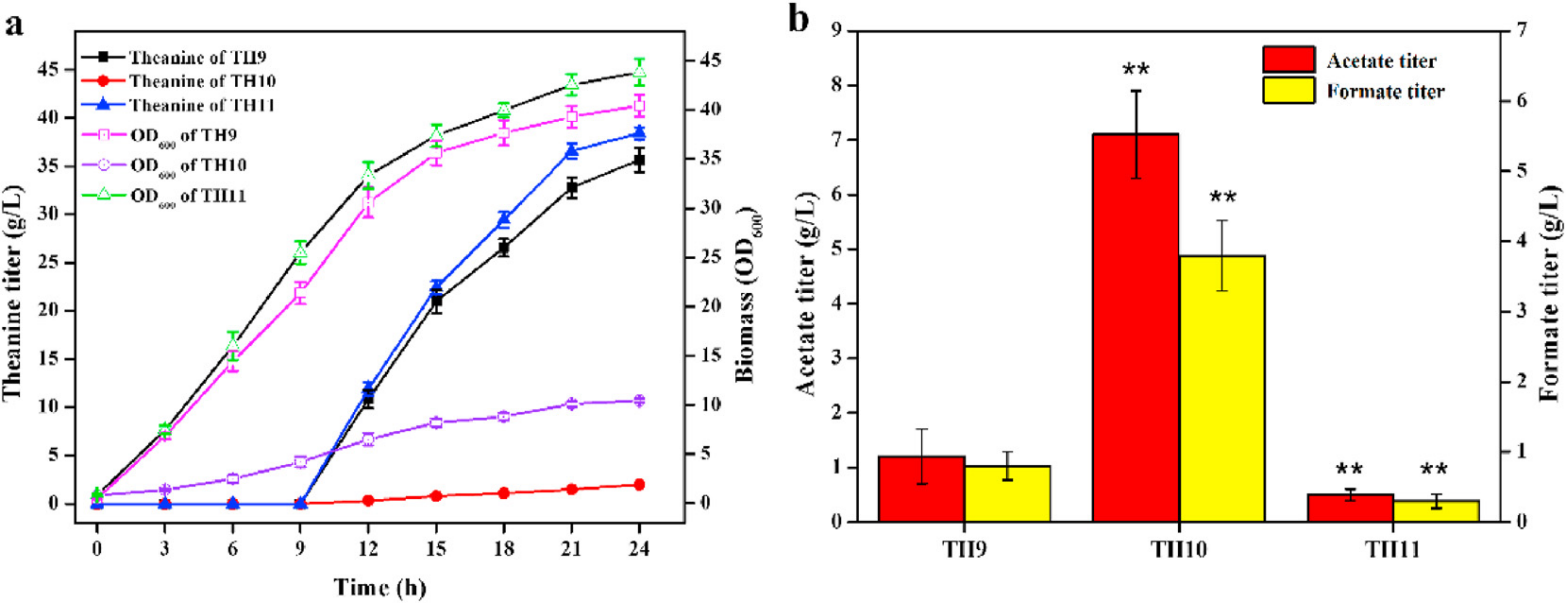
Effect of using phosphoenolpyruvate carboxykinase from *M. succiniciproducens* instead of phosphoenolpyruvate carboxylase (TH10) and complementing native phosphoenolpyruvate carboxylase (TH11) on theanine titer and biomass (**a**), and the formation of acetate and formate (**b**). Each experiment was repeated three times, and data are represented as the means of three replicates and bars represent the standard deviations. ∗, P ​< ​0.05 and ∗∗, P ​< ​0.01 indicate the significance level between the two engineered strain. e.g., TH10 v TH9, TH11 v TH10.

In the case of the ligation reaction of glutamate and ethylamine catalyzed by GMAS, a continuous supply of ATP is required, and the regeneration of ATP is essential for practical applications. In *E. coli*, phosphoenolpyruvate is converted to oxaloacetate mainly by PPC. However, energy contained in phosphoenolpyruvate is lost in this reaction with the release of inorganic phosphate (Yeongdeok et al., 2006). Rumen bacteria that produce succinate as the dominant product use an energy-conserving phosphoenolpyruvate carboxykinase (PCK) for oxaloacetate production (Zhang et al., 2009). Hence, we used heterogenous PCK instead of the native PPC in *E. coli* to improve the net production of ATP. The codon-optimized *pckA* from *M. succiniciproducens*, encoding PCK, was placed under the P_*trc*_ promoter and integrated into the *ppc* locus of TH9, generating strain TH10. As shown in Fig. 6a, TH10 could not grow well during the shake-flask fermentation. Only a few of theanine was accumulated in the cultures of TH10, while the acetate and formate titer were increased to 7.1 ​g/L and 3.8 ​g/L, respectively (Fig. 6b). These results indicated that the native PPC in *E. coli* was the most important anaplerotic enzyme for growth and maintenance under aerobic conditions.

Next, we complemented the native PPC and test the function of PCK from *M. succiniciproducens*. The *ppc* gene controlled by the native promoter was integrated into the pseudogene *gapC* locus of TH10, generating strain TH11. As shown in Fig. 6a, the cell growth of TH11 was completely restored, and the growth rate was even greater than that of TH9 during the supplementation of ethylamine hydrochloride. The theanine titer of TH11 (38.4 ​g/L) was improved by 7.6% compared with that of TH9, while the acetate and formate titer of TH9 were reduced by 58.3% and 62.5%, respectively. Introduction of heterogenous PCK can both increase the flux to oxaloacetate and save energy consumption, and thus provide a competitive advantage during theanine fermentation.

### 3.6 Optimization of ethylamine supplementation in a 5-L bioreactor

**Fig. 7:**
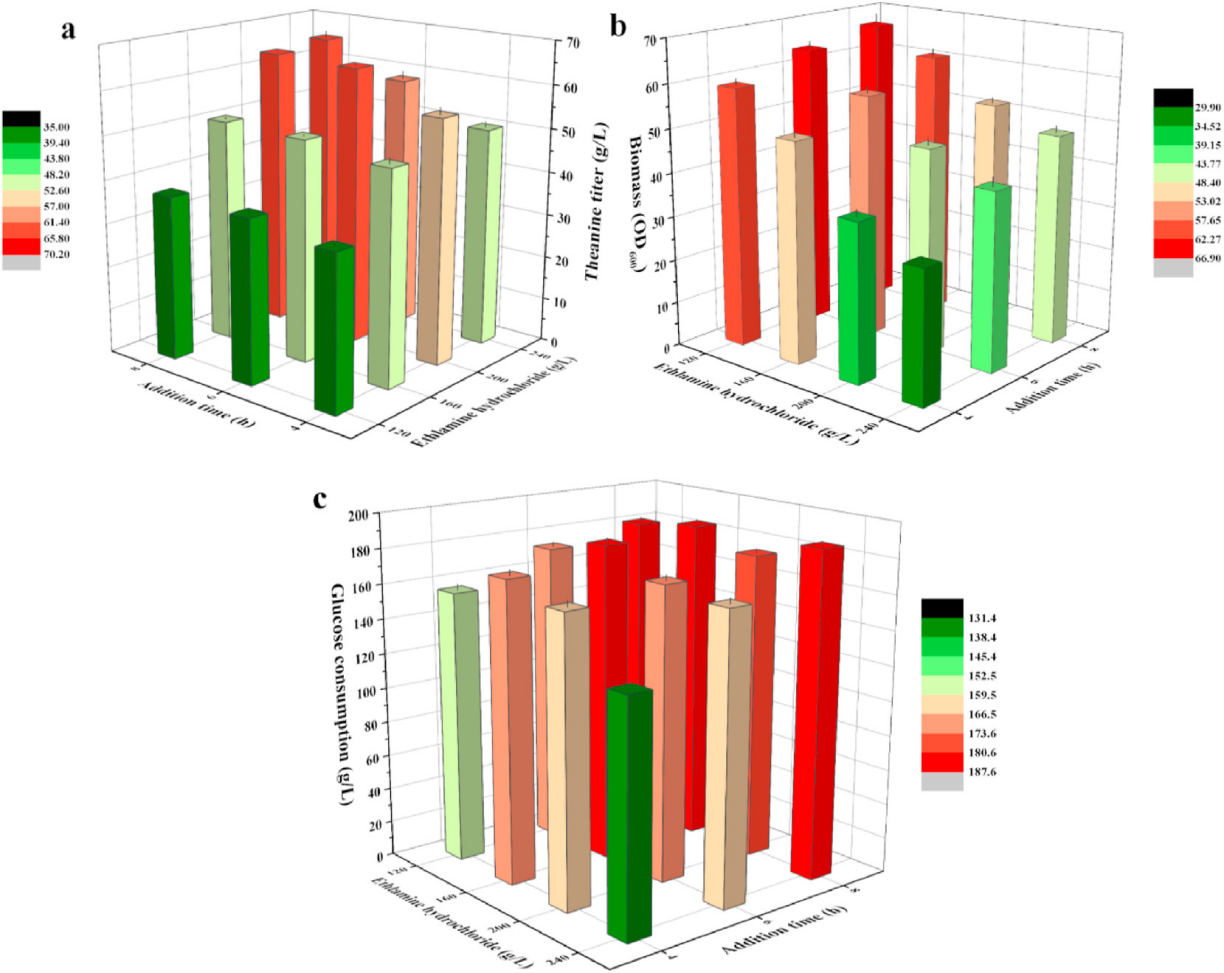
Effect of optimizing the addition time and concentration of ethylamine hydrochloride in a 5-L bioreactor on theanine titer (**a**), biomass (**b**), and glucose consumption (**c**). Each experiment was repeated three times, and data are represented as the means of three replicates and bars represent the standard deviations.

Theanine production with the recombinant strain *E. coli* TH11 was tested in a 5-L bioreactor. As an important precursor, the sufficient ethylamine in the fermentation system is crucial to increase the theanine yield and productivity. However, high concentration of ethylamine inhibited cell growth and glucose metabolism (Yamamoto et al., 2006, Yamamoto et al., 2008b), and thus affected the production of other precursors, i.e., glutamate and ATP. Hence, we adopted the fed-batch fermentation strategy and optimized the addition time and concentration of ethylamine hydrochloride. According to the growth curve of *E. coli* TH11, ethylamine hydrochloride (120–240 ​g/L) was initial added at different stages of the logarithmic growth phase (4–8 ​h) with a rate of 30 ​mL/h for 20 ​h. After fermentation, the volume of fermentation broth was fixed to 4 ​L. The detailed groupings were shown in Table S2 and the fermentation results were shown in Fig. 7. When the concentration of ethylamine hydrochloride was higher than 160 ​g/L, the theanine titer and biomass was obviously increased with the extension of addition time. These results indicated that appropriate prolongation of fermentation time was conducive to cell growth, and thus alleviated the toxicity of ethylamine. When the concentration of ethylamine hydrochloride was increased from 120 ​g/L to 200 ​g/L, the biomass was decreased, but more glucose was consumed for theanine production, and thus increased the theanine yield. The three most competitive groups were 6/200, 8/200 and 8/240, with theanine titer of 63.7 ​g/L, 64.1 ​g/L and 65.4 ​g/L, respectively. However, the productivity of group 6/200 (2.45 ​g/L/h) were 6.9% and 5.1% higher than that of group 8/200 and 8/240, respectively. Meanwhile, the theanine yield of group 6/200 (0.37 ​g/g glucose) were also higher than the other two groups. These results indicated that reasonable control of ethylamine feeding mode can effectively balance and optimize the ratio of biomass formation and theanine production. Comprehensive considering production cost, we chose group 6/200 as the best feeding mode of ethylamine hydrochloride.

**Fig. 8:**
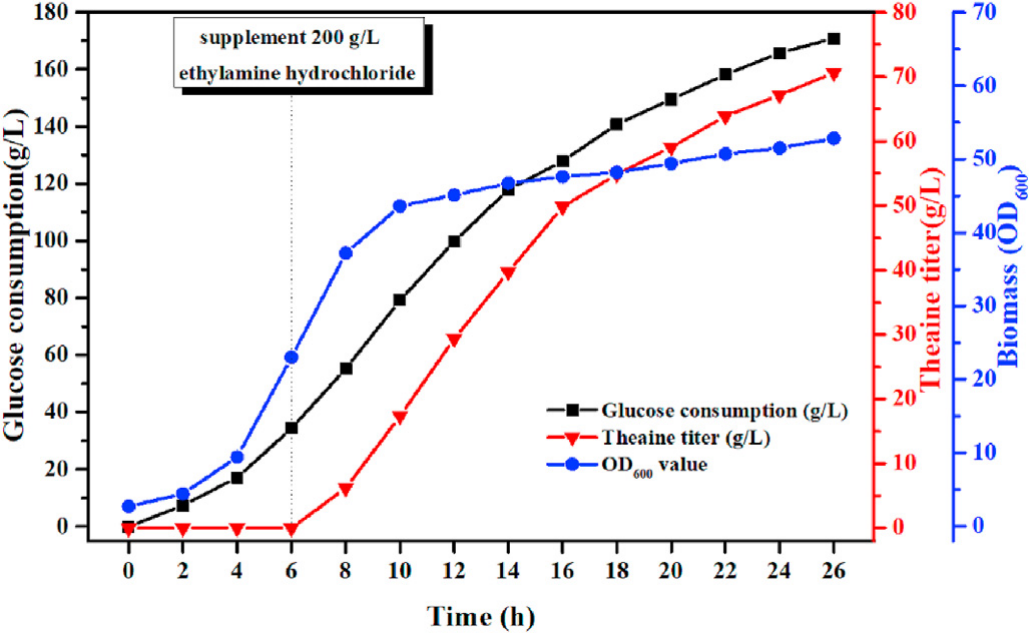
Fed-batch production of theanine in a 5-L bioreactor. Ethylamine hydrochloride (200 ​g/L) was automatically fed at a rate of 30 ​mL/h from 6 ​h to 26 ​h.

The production performance of *E. coli* TH11 under the best feeding mode of ethylamine hydrochloride was further tested and the results were shown in Fig. 8. The OD_600_ of the cultures peaked at ~45 ​at 10 ​h and then remained steady at this level. The extracellular theanine titer reached 70.6 ​g/L after 26 ​h of fermentation. The yield of theanine relative to glucose was 0.42 ​g/g, and the productivity was 2.72 ​g/L/h. In addition, less than 1 ​g/L acetate and formate were produced, while no other amino acid was detected as a by-product. Compared with our previous study (Ma et al., 2020), the theanine titer, productivity and yield of *E. coli* TH11 were much higher than that of *C. glutamicum* GDK-9 Δ*cgl1221*/pTuf-*gmas*_Mm_. Thus, the strain *E. coli* TH11 has greater potential to produce theanine in industrial-scale with the strategy of fed-batch ethylamine.

## 4 Discussion

GMAS has been successfully used as a key enzyme to catalyze the conversion of glutamate and ethylamine to theanine (Yamamoto et al., 2008a, 2008b; Liu et al., 2016; Ma et al., 2020). We previously expressed GMAS_*Mm*_ in a glutamate producer strain *C. glutamicum* GDK-9 using high-copy plasmid and achieved the fermentative production of theanine using ethylamine supplementation (Ma et al., 2020). Compared with enzyme catalyzed method (Yamamoto et al., 2008b), fermentation method is lack of competitiveness due to the low theanine titer (42 ​g/L) and yield (0.19 ​g/g glucose) (Table 2). Moreover, strain *C. glutamicum* GDK-9 were auxotrophic and genetically undefined, which hampered large-scale cultivation and further strain improvement.

**Table 2:** Production of L-theanine by using distinct methods.

Methods	Enzyme	Features	Main sources	L-theanine titer (g/L)	Conversion rate	Time (h)	References
Enzymatic catalysis	GMAS from *M. mays* No. 9	GMAS coupling with yeast sugar fermentation^a^	600 ​mM glutamate, 600 ​mM ethylamine hydrochloride, 300 ​mM glucose, 5 ​mM ATP	110 ​g/L	Molar yield of 100% on glutamate	48	Yamamoto et al., (2008b)
Enzymatic catalysis	GMAS from *M. mays* No. 9	GMAS coupling with PPK^b^	200 ​mM glutamate, 200 ​mM ethylamine hydrochloride, 75 ​mM polyP, 5 ​mM ATP	32 ​g/L	Molar yield of 93% on glutamate	5	Liu et al. (2016)
	PPK from *R. sphaeroides*						
Fermentation	GMAS from *M. mays* No. 9	Engineered *C. glutamicum*	1200 ​mM glucose, 260 ​mM ethylamine hydrochloride	42 ​g/L	Yield of 19.6% on glucose^c^	48	Ma et al. (2020)
Fermentation	GMAS from *P. aminovorans*	Engineered *E. coli*	930 ​mM glucose, 420 ​mM ethylamine hydrochloride	70.6 ​g/L	Yield of 42% on glucose^c^	26	This study

a Glucose was used as the energy source for the ATP-regenerating system during the yeast fermentation.

b Polyphosphate kinase (PPK) that catalyzes the reversible transfer of the phosphate molecular between ATP and inorganic polyphosphate (polyP).

c The yield is based on glucose consumed (wt%).

In this study, we report the development of a new engineered strain for high-level theanine production using *E. coli* W3110 as starting strain. GMAS_*Pa*_ was selected and introduced into the chromosome of *E. coli* to construct the theanine biosynthetic pathway, due to its higher activity compared with other GMAS. As the enzyme producing strain *P. aminovorans* JCM7685 was isolated from a contaminated soil (Urakami et al., 1990), it is possible to obtain better GMAS by screening and testing more methylotrophic bacteria from extremely polluted environment (Sharma et al., 2018). To ensure high-level expression of GMAS_*Pa*_, two regulation fragments were constructed, consisting of two different promoter sequences. The flask-level fermentation results demonstrated the highest theanine titer was achieved using one copies of T7 promoter controlled *gmas*_*pa*_, suggesting that T7 was more suitable for the expression of rate-limiting enzyme. Moreover, the promoter of *xylF* was inducible with xylose and combined with the T7 RNA polymerase to control gene expression (Nakashima et al., 2014). The *mlc* gene was mutated to keep glucose and xylose consumption at virtually identical rates (Nakashima and Tamura, 2012). These features are beneficial for utilizing the second-generation feedstock xylose. Therefore, the hemicellulose hydrolysate can be a potential carbon source for theanine fermentation.

Rational design of metabolic pathways based on current metabolic network models is a common method for engineering microorganisms for producing glutamate-derived compounds with maximum yield. Engineering strategies aiming at increasing precursor glutamate pool have been developed, such as tuning of the promoter of GDH gene (Jensen et al., 2015; Xu et al., 2018), and overexpressing the key enzymes CS, aconitase (ACN) and isocitrate dehydrogenase (IDH) (Chen et al., 2020b; Liu et al., 2018). GDH of *C. glutamicum* is reported to be constitutive enzymes, while in *E. coli* GDH synthesis is repressed by glutamate and is probably regulated by the CRP-cAMP complex (Bormann et al., 1992). Hence, we introduced GDH of *C. glutamicum* GDK-9, and overexpressed the native CS, ACN and IDH. However, ACN overexpression in strain TH7 showed no effect on theanine production and biomass, while IDH overexpression in strain TH7 increased the biomass rather than theanine production (data not shown). We subsequently rewired the TCA cycle by knocking out succinyl-CoA synthetase and introducing PYC of *C. glutamicum* GDK-9. Succinate and oxaloacetate could be replenished by the glyoxylate pathway and the constructed anaplerotic pathway. The flask-level fermentation results demonstrated the rewired TCA cycle could effectively redirect the pyruvate flux to theanine without affecting the normal cell growth.

The energy-conserving PCK was also be applied to improving theanine production from glucose in this study. The recruitment of PCK as the primary anaplerotic pathway for growth and theanine production in *E. coli* under aerobic conditions was unsuccessful. Previous studies showed that the K_m_ value of PCK for bicarbonate is 13 ​mM, whereas that of PPC for bicarbonate is 0.15 ​mM (Yeongdeok et al., 2006). Thus, PCK would be able to catalyze the reaction at a high concentration of bicarbonate. However, the divalent cation Mg^2+^ and Mn^2+^ are cofactors of GMAS (Yamamoto et al., 2008b), which is easy to precipitate with bicarbonate. We also tried to change the composition of medium by adding different amount of bicarbonate and reducing the amount of divalent cation for the cultivation of TH10, but the growth of TH10 in bicarbonate medium was still abolished (data not shown). Recently, evolutionary engineering has attracted more attention in the field of strain improvement (Stella et al., 2019). The glyoxylate shunt was evolved to function as the primary anaplerotic pathway in a PPC deficient mutant for aerobic succinate production (Li et al., 2013). Therefore, adaptive laboratory evolution method needs to be performed to increase the availability of energy-conserving PCK for growth and theanine production in the absence of PPC. The selection pressure of evolution is the insufficient supply of oxaloacetate, which resulted in the recruitment of PCK.

Through systematically optimizing the feeding mode of ethylamine, the recombinant strain *E. coli* TH11 was capable of producing theanine at a high titer of 70.6 ​g/L in a 5-L bioreactor, with a yield and productivity of 0.42 ​g/g glucose and 2.72 ​g/L/h, respectively (Table 2). To our knowledge, this is the first report regarding the pathway engineering of *E. coli* for fermentative production of theanine. Consist with our expectations, the recombinant strain *E. coli* TH11 can endogenously produce adequate glutamate and ATP, and completely convert ethylamine to theanine. However, it is still a challenge to construct the de novo synthetic pathway for fermentative production of theanine, because the decarboxylation of alanine to ethylamine is only discovered in tea plants. Although serine decarboxylase in *C. sinensis* exhibited alanine decarboxylation activity (Bai et al., 2019), this enzyme is difficult to be expressed effectively and correctly in *E.coli* (Ma et al., 2020). The structural similarity of alanine and serine raises the possibility that serine decarboxylase catalyzes alanine decarboxylation. Thus, enzyme engineering approaches must be used in the further to analyze the structure of serine decarboxylase from more sources and alter the substrate specificity for alanine decarboxylation. Overall, this study lays the foundation for industrial fermentative production of theanine from sugars and ethylamine. The metabolic and fermentative strategies have great application potential in developing efficient microbial cell factories for the production of N-alkylated amino acids.

## CRediT authorship contribution statement

**Xiaoguang Fan:** Conceptualization, Investigation, Writing - original draft. **Tong Zhang:** Investigation, Formal analysis. **Yuanqing Ji:** Investigation. **Jie Li:** Formal analysis. **Keyi Long:** Investigation. **Yue Yuan:** Investigation. **Yanjun Li:** Methodology, Writing - review & editing. **Qingyang Xu:** Methodology. **Ning Chen:** Resources. **Xixian Xie:** Conceptualization, Supervision, Funding acquisition.

## Declaration of competing interest

The authors declare that they have no known competing financial interests or personal relationships that could have appeared to influence the work reported in this paper.
