# Supplementary material for: Pathway engineering of Escherichia coli for one-step fermentative production of L-theanine from sugars and ethylamine
Source: Metab Eng Commun. 2020 Nov 6;11:e00151. doi: 10.1016/j.mec.2020.e00151 (PMC7677707; doi:10.1016/j.mec.2020.e00151)
Supplement: Multimedia component 1 [file mmc1.doc]

**Pathway engineering of *Escherichia coli* for one-step fermentative production of L-theanine from sugars and ethylamine**

Xiaoguang Fan ^a,b,#^, Tong Zhang ^b,#^, Yuanqing Ji ^b^, Jie Li ^b^, Keyi Long ^b^, Yue Yuan ^b^, Yanjun Li ^a,b^, Qingyang Xu ^a,b^, Ning Chen ^a,b^, Xixian Xie ^a,b,*^

**Table S1** Primers used for strain construction

| Primers | Sequence (5’→ 3’) |
| --- | --- |
| UP-*yghX*-S | GCGCAACGTAGAACAGGAATT |
| UP-P*_trc_*-*yghX-*A | AATTGTTATCCGCTCACAATTCCACACATTATACGAGCCGGATGATTAATTGTCAAGATTGAAGCGCCTTTACTACTCC |
| DN-T*_trc_*-*yghX*-S | CTGGGCCTTTCGTTTTATCTGTTGTTTGTCGGTGAACGCTCTCCTGAGTAGGACAAATGTCATAGTAATCCAGCAACTCTTGTG |
| DN-*yghX*-A | GAGCAGGTATTTACGTGAACCG |
| P*_trc_*-*gmas_Mu_*-S | TCCGGCTCGTATAATGTGTGGAATTGTGAGCGGATAACAATTTCACACAGGAAACAGACCATGAGCCCGAGCGAAGCCCAGCAGT |
| T*_trc_*-*gmas_Mu_*-A | ACAAACAACAGATAAAACGAAAGGCCCAGTCTTTCGACTGAGCCTTTCGTTTTATTTGTTAAAAAAATTCCAGATAAGAATTC |
| P*_trc_*-*gmas_Me_*-S | TCCGGCTCGTATAATGTGTGGAATTGTGAGCGGATAACAATTTCACACAGGAAACAGACATGAAGTCCCTCGAGGAAGCTC |
| T*_trc_*-*gmas_Me_*-A | ACAAACAACAGATAAAACGAAAGGCCCAGTCTTTCGACTGAGCCTTTCGTTTTATTTGTTAGTAAAACTGCACGTAACGGTTG |
| P*_trc_*-*gmas_Mm_*-S | TCCGGCTCGTATAATGTGTGGAATTGTGAGCGGATAACAATTTCACACAGGAAACAGACCATGAAAAGTCTGGAAGAAGCTCAGA |
| T*_trc_*-*gmas_Mm_*-A | ACAAACAACAGATAAAACGAAAGGCCCAGTCTTTCGACTGAGCCTTTCGTTTTATTTGTTAATAGAACTGCACATAGCGATTGA |
| P*_trc_*-*gmas_Pa_*-S | TCCGGCTCGTATAATGTGTGGAATTGTGAGCGGATAACAATTTCACACAGGAAACAGACCATGACCGATCTGGCCGAATTTGCCC |
| T*_trc_*-*gmas_Pa_*-A | ACAAACAACAGATAAAACGAAAGGCCCAGTCTTTCGACTGAGCCTTTCGTTTTATTTGTTAAATATCTAAAGTGTGATCGCGT |
| sgRNA-*yghX*-S | AGTCCTAGGTATAATACTAGTGGTGCCTGACGACCATAAAAGTTTTAGAGCTAGAA |
| sgRNA-*yghX*-A | TTCTAGCTCTAAAACTTTTATGGTCGTCAGGCACCACTAGTATTATACCTAGGACT |
| UP-*yeeP*-S | GGTCAGGAGGTAACTTATCAGCG |
| UP-P*_trc_*-*yeeP*-A | AATTGTTATCCGCTCACAATTCCACACATTATACGAGCCGGATGATTAATTGTCAAATGGCAGGGCTCCGTTTT |
| DN-T*_trc_*-*yeeP*-S | AAAGACTGGGCCTTTCGTTTTATCTGTTGTTTGTCGGTGAACGCTCTCCTGAGTAGGACAAATGAACTGGATTTTCTTCTGAACCTGT |
| DN-*yeeP*-A | ACGATGTCAGCAGCCAGCA |
| sgRNA-*yeeP*-S | AGTCCTAGGTATAATACTAGTACAGAATATTCGCGAAAAAAGTTTTAGAGCTAGAA |
| sgRNA-*yeeP*-A | TTCTAGCTCTAAAACTTTTTTCGCGAATATTCTGTACTAGTATTATACCTAGGACT |
| UP-*mbhA*-S | GCCAGCACGAACATAATCCC |
| UP-P*_trc_*-*mbhA*-A | AATTGTTATCCGCTCACAATTCCACACATTATACGAGCCGGATGATTAATTGTCAACACGGTGGCAGGTTTTGG |
| DN-T*_trc_*-*mbhA*-S | CTGGGCCTTTCGTTTTATCTGTTGTTTGTCGGTGAACGCTCTCCTGAGTAGGACAAATGACCAAAAGTGCGTCCGATAC |
| DN-*mbhA*-A | CGGCGTAATCACAAACTGGC |
| sgRNA-*mbhA*-S | AGTCCTAGGTATAATACTAGTTTCCAAAAATCCGCAAAGCGGTTTTAGAGCTAGAA |
| sgRNA-*mbhA*-A | TTCTAGCTCTAAAACCGCTTTGCGGATTTTTGGAAACTAGTATTATACCTAGGACT |
| UP-*lacI*-S | ACAACAACTGGCGGGCAAAC |
| UP-P*_xylF_*-*lacI*-A | CGAGCGCACTTGTGAATTATCTCCGCCGAGACAGAACTTAATGGG |
| DN-T*_xylF_*-*lacI*-S | aaataccttgatactgtgccggcaggtagcagagcgggtaaactggctcggatt |
| DN-*lacI*-A | ggatttccttacgcgaaatacg |
| P*_xylF_*-T7RNAP-S | cccattaagttctgtctcggcggagataattcacaagtgtgcgctcg |
| T*_xylF_*-T7RNAP-A | aatccgagccagtttacccgctctgctacctgccggcacagtatcaaggtattt |
| sgRNA-*lacI*-S | agtcctaggtataatactagtcagccgatagcggaacgggagttttagagctagaa |
| sgRNA-*lacI*-A | ttctagctctaaaactcccgttccgctatcggctgactagtattatacct aggact |
| UP-P*_T7_*-*yghX-*A | TAAAGTTAAACAAAATTATTTCTAGACCCTATAGTGAGTCGTATTAGATTGAAGCGCCTTTACTACTCC |
| DN-T*_T7_*-*yghX*-S | TGGGGCCTCTAAACGGGTCTTGAGGGGTTTTTTGGTCATAGTAATCCAGCAACTCTTGTG |
| P*_T7_*-*gmas_Pa_*-S | TAGGGTCTAGAAATAATTTTGTTTAACTTTAAGAAGGAGATATACCATGACCGATCTGGCCGAATTTGCCC |
| T*_T7_*-*gmas_Pa_*-A | AGACCCGTTTAGAGGCCCCAAGGGGTTATGCTAGTTAAATATCTAAAGTGTGATCGCGT |
| UP-P*_T7_*-*yeeP*-A | TAAAGTTAAACAAAATTATTTCTAGACCCTATAGTGAGTCGTATTAATGGCAGGGCTCCGTTTT |
| DN-T*_T7_*-*yeeP*-S | TGGGGCCTCTAAACGGGTCTTGAGGGGTTTTTTGGAACTGGATTTTCTTCTGAACCTGT |
| UP-*ylbE*-S | ACCCAACCTTACGCAACCAG |
| UP-P*_trc_*-*ylbE*-A | AATTGTTATCCGCTCACAATTCCACACATTATACGAGCCGGATGATTAATTGTCAATTGTTCGATAACCGCAGCAT |
| DN-T*_trc_*-*ylbE*-S | AAAGACTGGGCCTTTCGTTTTATCTGTTGTTTGTCGGTGAACGCTCTCCTGAGTAGGACAAATCGCTGGCGTGCTTTGAA |
| DN-*ylbE*-A | GGCGTAACTCAGCAGGCAG |
| P*_trc_*-*gltA*-S | TCCGGCTCGTATAATGTGTGGAATTGTGAGCGGATAACAATTTCACACAGGAAACAGACCATGGCTGATACAAAAGCAAAACTC |
| T*_trc_*-*gltA*-A | CACCGACAAACAACAGATAAAACGAAAGGCCCAGTCTTTCGACTGAGCCTTTCGTTTTATTTGTTAACGCTTGATATCGCTTTTAAAG |
| sgRNA-*ylbE*-S | AGTCCTAGGTATAATACTAGTGCGCCTGTAAACACTCCGCAGTTTTAGAGCTAGAA |
| sgRNA-*ylbE*-A | TTCTAGCTCTAAAACTGCGGAGTGTTTACAGGCGCACTAGTATTATACCTAGGACT |
| P*_trc_*-*cgl2079*-S | TCCGGCTCGTATAATGTGTGGAATTGTGAGCGGATAACAATTTCACACAGGAAACAGACCATGACAGTTGATGAGCAGGTCTCTA |
| T*_trc_*-*cgl2079*-A | ACAAACAACAGATAAAACGAAAGGCCCAGTCTTTCGACTGAGCCTTTCGTTTTATTTGTTAGATGACGCCCTGTGCC |
| UP-*sucCD*-S | CAACTTTTTGCCCGCTATGG |
| UP-*sucCD*-A | AGTTGCTGGATTACTATGACCCTAGAAGAAATCAACCAGCGCATCAGAAAGTCTCCTGTGCAT CCAGTTTACCTTCCAGACCCAG |
| DN-*sucCD*-S | ATGCACAGGAGACTTTCTGATGCGCTGGTTGATTTCTTCTAGGGTCATAGTAATCCAGCAACT ATTATCACCATCACTGAAGGCATC |
| DN-*sucCD*-A | GGCGAGGGCTATTTCTTATTACA |
| sgRNA-*sucCD*-S | AGTCCTAGGTATAATACTAGTCCCGCTGGTCATCACCAAACGTTTTAGAGCTAGAA |
| sgRNA-*sucCD*-A | TTCTAGCTCTAAAACGTTTGGTGATGACCAGCGGGACTAGTATTATACCTAGGACT |
| UP-*yghE*-S | GTCAGGCACTGGCGAAAGAT |
| UP-P*_trc_*-*yghE*-A | AATTGTTATCCGCTCACAATTCCACACATTATACGAGCCGGATGATTAATTGTCAACGCAAGCCATAAACCCACA |
| DN-T*_trc_*-*yghE*-S | CTGGGCCTTTCGTTTTATCTGTTGTTTGTCGGTGAACGCTCTCCTGAGTAGGACAAATTTCCGACATCGAAATGCGT |
| DN-*yghE*-A | AGGCGTTGTTGTGGCAGATT |
| P*_trc_*-*cgl0689*-S | TCCGGCTCGTATAATGTGTGGAATTGTGAGCGGATAACAATTTCACACAGGAAACAGACCGTGTCGACTCACACATCTTCAACG |
| T*_trc_*-*cgl0689*-A | ACAAACAACAGATAAAACGAAAGGCCCAGTCTTTCGACTGAGCCTTTCGTTTTATTTGTTAGGAAACGACGACGATCAAGTC |
| sgRNA-*yghE*-S | AGTCCTAGGTATAATACTAGTCATTACCACTTATGGCGAACGTTTTAGAGCTAGAA |
| sgRNA-*yghE*-A | TTCTAGCTCTAAAACGTTCGCCATAAGTGGTAATGACTAGTATTATACCTAGGACT |
| UP-*ppc*-S | TGGTGAACTACTGTACCGAAGCG |
| UP-P*_trc_*-*ppc*-A | AATTGTTATCCGCTCACAATTCCACACATTATACGAGCCGGATGATTAATTGTCAATGAGTCACTGTCGGTCGGATAAG |
| DN-T*_trc_*-*ppc*-S | CTGGGCCTTTCGTTTTATCTGTTGTTTGTCGGTGAACGCTCTCCTGAGTAGGACAAATTGGCTGGCGGAATACTATGAC |
| DN-*ppc*-A | CTGTCTTGAAAAAATATCGCCG |
| P*_trc_*-*pckA_MS_*-S | TCCGGCTCGTATAATGTGTGGAATTGTGAGCGGATAACAATTTCACACAGGAAACAGACCATGACAGATCTTAATCAATTAACTCAAGA |
| T*_trc_*-*pckA_MS_*-A | ACAAACAACAGATAAAACGAAAGGCCCAGTCTTTCGACTGAGCCTTTCGTTTTATTTGTTATGCTTTAGGACCGGCAG |
| sgRNA-*ppc*-S | AGTCCTAGGTATAATACTAGTACGATAAGATGGGGTGTCTGGTTTTAGAGCTAGAA |
| sgRNA-*ppc*-A | TTCTAGCTCTAAAACCAGACACCCCATCTTATCGTACTAGTATTATACCTAGGACT |
| UP-*gapC*-S | TGGGAAGAAACCACGAAACTC |
| UP-*gapC*-A | CCCAAGTAACACCAAAGGTGTAGTGTTTCAGCAGGTAGGCGAGA |
| DN-*gapC*-S | AGGTATGCGTAATACCGGCTAAAAAACGGTCGCCTGGTACG |
| DN-*gapC*-A | TTATCCGCCGACATTGCTG |
| *ppc*-S | TCTCGCCTACCTGCTGAAACACTACACCTTTGGTGTTACTTGGG |
| *ppc*-A | CGTACCAGGCGACCGTTTTTTAGCCGGTATTACGCATACCT |
| sgRNA-*gapC*-S | AGTCCTAGGTATAATACTAGTCTATACTGGCACCCAGTCACGTTTTAGAGCTAGAA |
| sgRNA-*gapC*-A | TTCTAGCTCTAAAACGTGACTGGGTGCCAGTATAGACTAGTATTATACCTAGGACT |

**Table S2** Experimental grouping for the feeding mode of ethylamine hydrochloride

| Concentration  Addition  time | 120 g/L | 160 g/L | 200 g/L | 240 g/L |
| --- | --- | --- | --- | --- |
| 4 h | 4/120 | 4/160 | 4/200 | 4/240 |
| 6 h | 6/120 | 6/160 | 6/200 | 6/240 |
| 8 h | 8/120 | 8/160 | 8/200 | 8/240 |


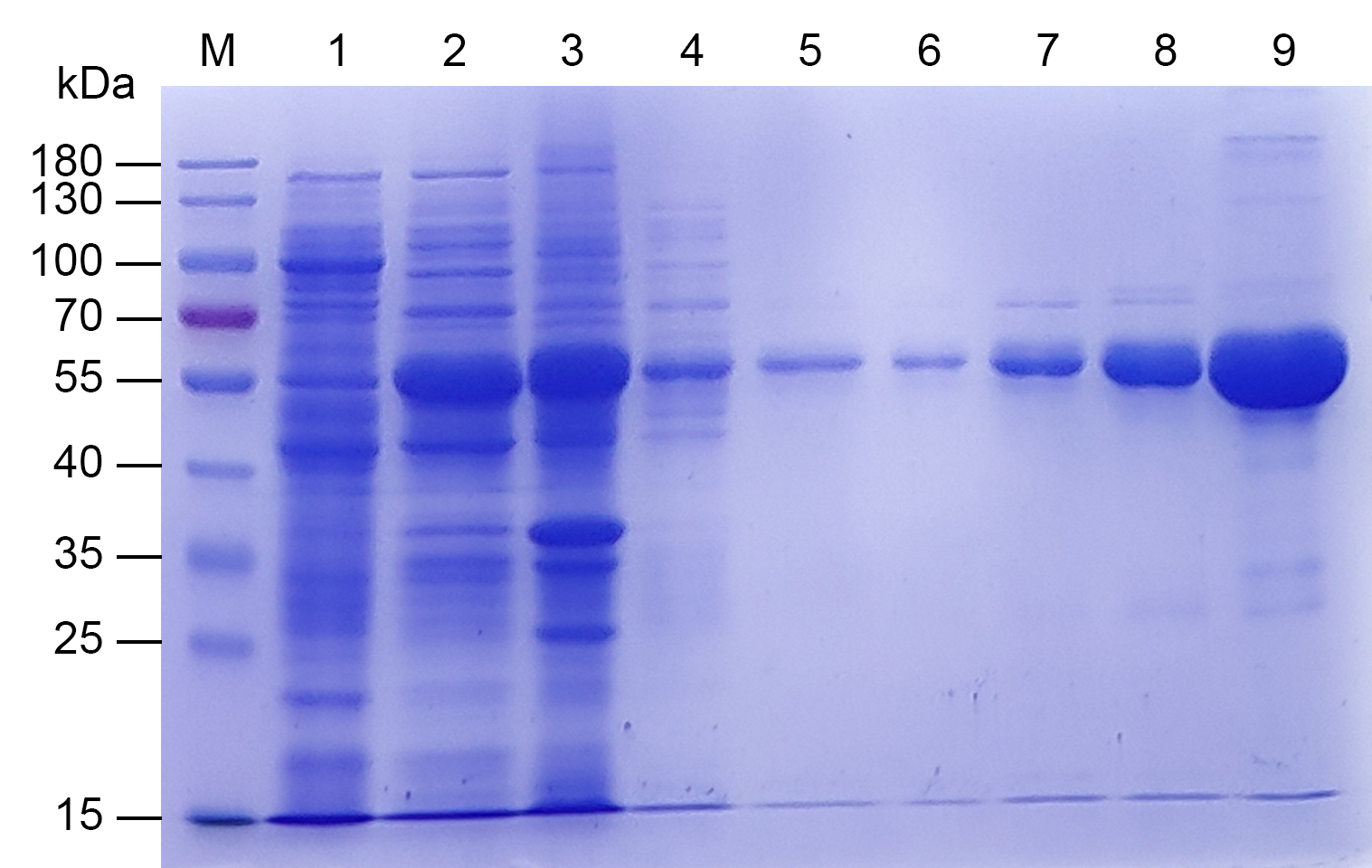


**Figure S1** SDS-PAGE analysis of GMAS*_pa_* expressed in *E. coli* BL21(DE3). Lane M: protein markers; Lanes 1: Supernatant from BL21(DE3) harboring empty plasmid; Lanes 2 and 3: Supernatant and precipitate of GMAS*_pa_*; Lanes 4-9: Purified enzyme of GMAS*_pa_* using 25, 50, 75, 100, 150, 300 mM imidazole elution.


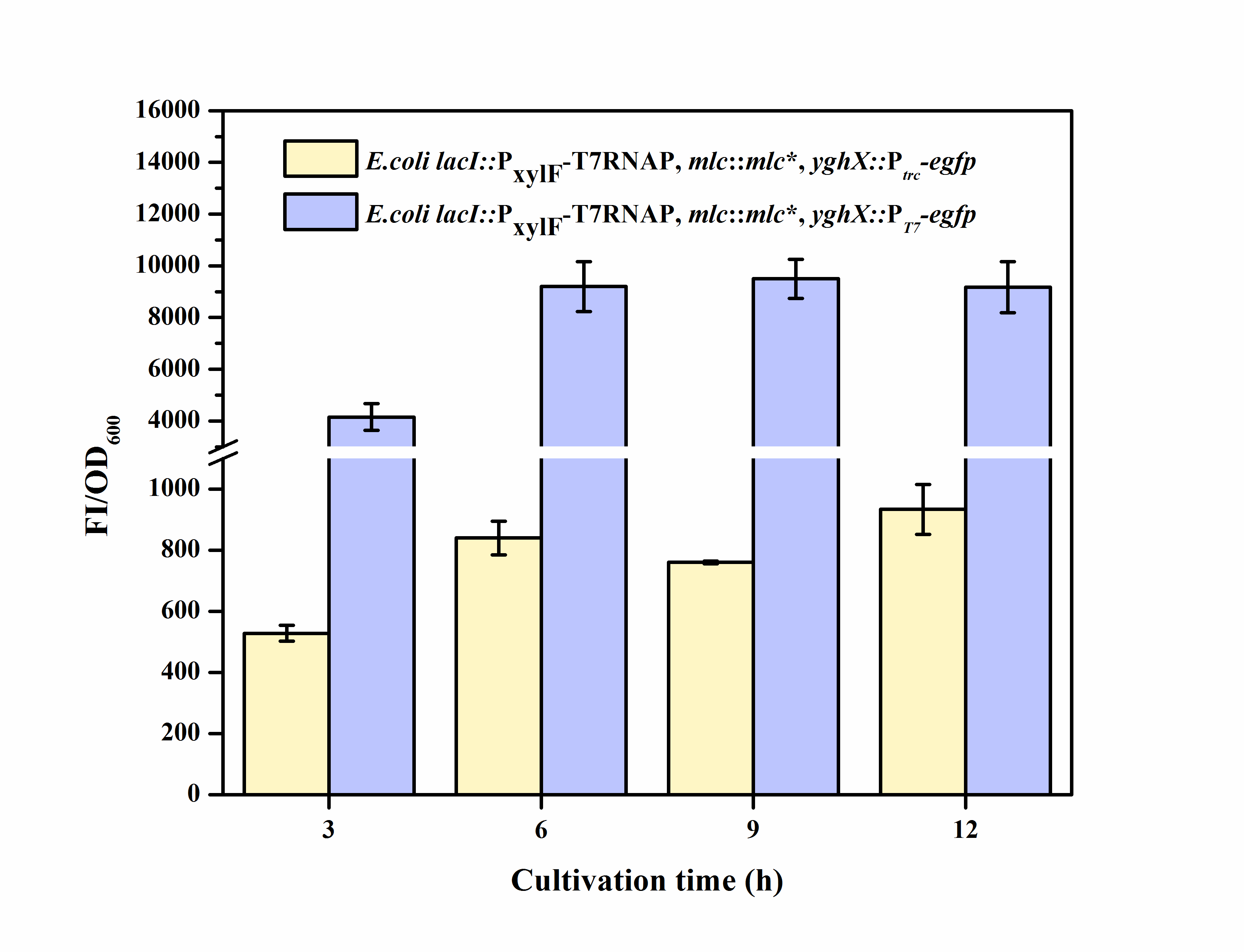


**Figure S2** Evaluation of expression level of P*_T7_* or P*_trc_* controlled green fluorescence protein gene. 5 g/L xylose were initially added to LB medium as inducers. The data are presented as mean ± SD from triplicate experiments.

Promoters and heterologous genes sequences used in this study.

T7：

TTGACAATTAATCATCCGGCTCGTATAATGTGTGGAATTGTGAGCGGATAACAATTTCACACAGGAAACAGACC

Trc：

CAAATAAAACGAAAGGCTCAGTCGAAAGACTGGGCCTTTCGTTTTATCTGTTGTTTGTCGGTGAACGCTCTCCTGAGTAGGACAAAT

*gmas_Mu_*：ATGAGCCCGAGCGAAGCCCAGCAGTTTTTAAAAGAAAATCAAGTTAAATATATTTTAGCACAGTTTGTGGATATCCACGGTAGCGCCAAGACCAAGAGCGTGCCCGCTGAACACTACAAAACCGTGGTGACAGACGGTGCCGGCTTTGCCGGCTTCGCCATTTGGGGCATGGGCATGACCCCTAATGTGGATGCCGACTACATGGCCGTTGGTGATGCAAGCACACTGAGTCTGGTGCCGTGGCAGCCGGGCTATGCACGTATTGCTTGTGATGGTCACACCCATGGCAAGCCGCACGAATACGATACCCGCGTGGTTCTGAAGAAGCAGTTAGAGCAGATTACCGCCCGCGGCTGGACCTTCTTCACCGGCATGGAACCGGAATTTTCTTTACTGCGCAAAGTGGAAGGCAAACTGCTGCCGGCCGATCCGGGTGATACTTTAAGCAAACCGTGCTACGATTACAAGGGTCTGAGTCGCGCCCGCGTGTTTTTAGAACGTCTGAGCGAATCTTTACGTAGTGTTGGCATCGACGTGTACCAGATTGACCATGAGGACGCCAATGGTCAGTTTGAGATTAATTACACCTTCACCGATGCCTTAACCAGTTGCGACCACTACACCTTCTTCAAAATGGGCGCAGCCGAGATTGCCGCAGAACTGGGTCTGATCTGCAGCTTTATGCCGAAGCCGTTCAGTAACCGTCCGGGTAATGGTTTACACATGCATATGAGCATCGGCGACGGCAAGCGCAATTTATTCGAGGATAAGAGCGATAAGCACGGTCTGGCTTTAAGTAAACTGGCATATCACTGGGCAGCCGGTCTGCTGAAACATGCCCCGGCACTGGCCGCATTATGCTGCCCGACCGTTAATAGCTATAAACGTCTGGTTGTTGGCCGCAGTCTGACCGGCGCAACATGGGCACCGGCCTACATTTGCTATGGCGGCAACAATCGCAGTGGTATGATCCGCAGTCCGGGTGGTCGTCTGGAACTGCGTCTGCCGGATGCAAGCTGCAATGCATATTTAGCAACCGCCGCCGTGATTGCAGCTGGTATGGACGGTGTGATCAATGAACTGGATCCGGGCGCCCCGCAGAATGACAATCTGTATGAGTATAGTCAAGCTCAACTGGATGCAGCCGGTATCAAAGTGCTGCCGCAGAATCTGCACGAAGCTTTACTGGCTTTAGAGAAAGATGAAGTGATTCGCAGTGCTTTAGGCCCGGTTGTTGATGAATTTTTACGTTTAAAACATATGGAGTGGGTGGAGTACATGCGCCACGTGAGCGATTGGGAAGTGAATTCTTATCTGGAATTTTTTTAA

*gmas_Me_*：

ATGGCTCACGATCTGGAAACTGCTGCTCGCGAGCGTGGCATCAAGTACTTCCTCGTGTCCTACACTGACCTCTTCGGCACTCAGCGTGCAAAGCTGGTGCCAGCAGCTGCAATCGGCTCCACTTGCCGCAACGGTGCTGGCTTCGCTGGCTTCGCTACTTGGCTGGATATGTCCCCAGCAGATGCAGATCTGCTGGCTATGCCAGATGCAGATGGCCTCATCCAGCTGCCATGGAAACCAGAAGTCGGCTGGCTGCCAGCAGATCTGGTGATGAACGGCAAGGCTGTGGAGCAAGGCCCTCGCAACATTCTGAAGCGTCTGATCAAGGAAGCAGCTGAAGAGGGCCTCCAAATGAAGTCCGGCGTGGAGTGCGAGTTTTTTCTGATCACCCCATGCGGTTCCGAGCCAGCTGATACCGCTGACAAGCAGACCAAACCATGCTACGACCAGTCCGCACTGATGCGCCGCTACGAAGTGATCACCGAAATCTGCGACGCTATGCTGTCCCTCGGCTGGAAGCCTTACCAGAATGACCACGAGGATGCAAACGGCCAGTTCGAGATGAACTGGGATTACGACGACGCTCTCATCACCGCAGATCGTCACGCATTCTTTAAGTACATGACCCGCTCCATCGCTGAAAAGCACGGCTTTCGCGCAACCTTCATGCCTAAGCCATTCATGGATCTGACTGGCTCCGGTTGTCATGCTCACGTCTCTCTGTGGCGCGATGGCCAGAACGTCTTCGCAGATCGCTCCGACGAGGTCGGTCTCTCCCAGATCGGCTACCACTTCATTGGCGGCCTCATCCACTCCGCAGACGCACTGGCTGCACTCACCAATCCATGCGTCAATTCCTACAAGCGCATCAACGCTCCTCGCACCACCTCCGGTGCTACTTGGGCTCCTAACACCGTGACTTACACCGGCAACAACCGCACCCATATGATCCGTATCCCAGATGGTGGCCGTTTCGAATTCCGTCTGGCAGACGGCGCAGCAAACCCTTATCTGCTGCAAGCTGGTCTGCTGGCTGCTGGTCTGGATGGTATTCGCCAGCGCCGCGATCCGGGCCAACGCCTCGACATCAACATGTACACCGATGGTCACACCGTGGAGGGCGTGAAGCGTCTGCCACTGAATCTGCTGGACGCACTGCGCGCACTGGAAGCTTCCCCAGTCCTCAACGAAGCACTCGGTGCTTTCGTCCCATCCTACCTCAAGCTGAAGCGCCAAGAATGGGATGACTACTGTCGCCATCTGACCCAGTGGGAACGCGATACCACTCTCGACTGCTAA

*gmas_Mm_*：

ATGAAAAGTCTGGAAGAAGCTCAGAAGTTCCTGGAGGACCACCATGTGAAATACGTGCTGGCCCAGTTTGTGGACATCCACGGCGTGGCAAAAGTTAAAAGCGTGCCGGCAAGCCATCTGAACGATATTCTGACCACCGGTGCAGGCTTTGCCGGTGGTGCCATTTGGGGTACCGGTATTGCCCCGAATGGTCCGGATTACATGGCCATTGGCGAACTGAGCACACTGAGCCTGATTCCGTGGCAACCGGGCTATGCACGTCTGGTGTGTGATGGTCATGTGAATGGCAAGCCGTACGAGTTTGACACCCGCGTTGTTCTGAAACAGCAGATCGCACGTCTGGCCGAGAAAGGCTGGACCCTGTATACCGGTCTGGAGCCTGAGTTTAGCCTGCTGAAGAAAGACGAGCATGGCGCAGTGCACCCGTTCGATGACAGCGATACACTGCAGAAACCGTGCTACGACTACAAAGGCATCACCCGTCATAGCCCGTTCCTGGAAAAGCTGACCGAAAGCCTGGTGGAGGTTGGCCTGGATATCTACCAGATCGATCACGAGGACGCAAATGGCCAGTTCGAAATCAATTACACCTATGCCGATTGCCTGAAAAGTGCCGACGACTATATTATGTTTAAGATGGCAGCAAGCGAGATTGCCAACGAACTGGGCATCATCTGCAGCTTCATGCCGAAACCGTTCAGTAACCGCCCTGGTAATGGCATGCATATGCACATGAGCATTGGCGACGGCAAGAAAAGCCTGTTTCAGGACGACAGCGATCCGAGTGGCCTGGGTCTGAGCAAGCTGGCCTATCATTTCCTGGGCGGTATTCTGGCACATGCACCGGCACTGGCAGCCGTTTGTGCCCCTACCGTGAACAGCTACAAGCGCCTGGTTGTTGGTCGTAGCCTGAGTGGTGCCACCTGGGCCCCGGCATACATCGCATACGGCAACAACAACCGTAGCACACTGGTTCGCATCCCGTATGGCCGTCTGGAACTGCGTCTGCCGGACGGTAGCTGTAACCCGTACCTGGCCACCGCAGCAGTGATTGCAGCCGGCTTAGATGGTGTTGCACGCGAATTAGACCCGGGTACCGGTCGTGATGATAATCTGTACGATTACAGTCTGGAACAGCTGGCCGAGTTTGGCATCGGCATTCTGCCGCAGAATTTAGGTGAAGCCCTGGACGCCCTGGAAGCCGATCAGGTGATTATGGATGCCATGGGTCCTGGCCTGAGCAAGGAATTCGTGGAGCTGAAGCGTATGGAGTGGGTTGACTATATGCGCCACGTGAGCGACTGGGAAATCAATCGCTATGTGCAGTTCTATTAA

*gmas_Pa_*：

ATGACCGATCTGGCCGAATTTGCCCGCGAAAAAGGCGTGAAATATTTTATGGTGAGCTATACCGATTTAGTGGGCGCCCAGCGCGCAAAACTGGTTCCGACCTACATGATCAACAACGTTGTGAGCGGCGGCGCCGGTTTTGCCGGCTTTGCTGGTGGCTTTGTTTTAACCCCGGCACATCCGGATATGTTAGGTATGCCGGATGCCGATACCGTTATCCAACTGCCGTGGAAACCGGAAGTTGCATGGGTGGCAGCCAACCCGGCAATGTATGATAGCCCGCTGCCGCAAGCTCCGCGTAATGTGCTGCGTAATGTGATTGCAGAGATGGAAAAGGAAGGTTTACGCATCAAAACCGGCGTTGAACCGGAGTTCTTCTTTCTGACTCCGGAAGGCGATCGTATTGCCGATACCCGCGATACCGCCGCCAAACCGTGCTACGATCAGCAAGCTATTATGCGTCGTTACGATGTGATCAGCGAGGTGAGCGACTACATGATTGAACTGGGCTGGGAACCGTATCAGAGTGACCATGAAGACGCCAACGGTCAGTTCGAGATGAACTGGAAGTATGATGATTCTTTAGCAACCGCCGATAAGCTGGCCTTTTTTAAATTTATGATGAAAAGCGTTGCCGAAAAGCATGGTCTGCGCGTGACCTTCATGCCGAAACCGTTCTTAGAGCTGACCGGTAGCGGCATGCATGCCCACATTAGCGGCTGGAGTCTGGATGGCAAAACCAACGCCTTCTACGATGGCAACGATGAGCTGGGTCTGAGCGAAGTGGGTCACCATTTTCTGGGCGGCATTATGAAGCATGCCAGCGCACTGGCCGCCATTACCAATCCGACCATCAATAGCTATAAACGCATTAACGCACCGCGTAGCAGTAGCGGTGCAACTTGGGCCCCGAATAGCGTGACTTGGAGCGGCGACAACCGCACCCATCTGGTGCGTGTTCCGGGCAAGGGTCGTATTGAACTGCGTTTACCGGATGGTGCCAGCAACCCGTATCTGTTACACGCCGTGATCATGGCCGCTGGTCTGGATGGTATTCGCCACAAGTGTGATCCGGGCAAGCGTCTGGACATTGATATGTACGCCGACGGCCATATGGTGAAAGATGCCCCGAAGCTGCCGCTGAATTTACTGGATGCCATTCGTGCCTTTGATCAGAACACCGAGCTGAAAGCCGCTTTAGGTGAAGAATTTAGCGCCAGCTTCATCGAGATGAAAATGAAAGAGTGGAATGCCTACGCAAGCCATCTGACCCAGTGGGAACGCGATCACACTTTAGATATTTAA

*cgl0689*:

GTGTCGACTCACACATCTTCAACGCTTCCAGCATTCAAAAAGATCTTGGTAGCAAACCGCGGCGAAATCGCGGTCCGTGCTTTCCGTGCAGCACTCGAAACCGGTGCAGCCACGGTAGCTATTTACCCCCGTGAAGATCGGGGATCATTCCACCGCTCTTTTGCTTCTGAAGCTGTCCGCATTGGTACCGAAGGCTCACCAGTCAAGGCGTACCTGGACATCGATGAAATTATCGGTGCAGCTAAAAAAGTTAAAGCAGATGCCATTTACCCGGGATACGGCTTCCTGTCTGAAAATGCCCAGCTTGCCCGCGAGTGTGCGGAAAACGGCATTACTTTTATTGGCCCAACCCCAGAGGTTCTTGATCTCACCGGTGATAAGTCTCGCGCGGTAACCGCCGCGAAGAAGGCTGGTCTGCCAGTTTTGGCGGAATCCACCCCGAGCAAAAACATCGATGAGATCGTTAAAAGCGCTGAAGGCCAGACTTACCCCATCTTTGTGAAGGCAGTTGCCGGTGGTGGCGGACGCGGTATGCGTTTTGTTGCTTCACCTGATGAGCTTCGCAAATTAGCAACAGAAGCATCTCGTGAAGCTGAAGCGGCTTTCGGCGATGGCGCGGTATATGTCGAACGTGCTGTGATTAACCCTCAGCATATTGAAGTGCAGATCCTTGGCGATCACACTGGAGAAGTTGTACACCTTTATGAACGTGACTGCTCACTGCAGCGTCGTCACCAAAAAGTTGTCGAAATTGCGCCAGCACAGCATTTGGATCCAGAACTGCGTGATCGCATTTGTGCGGATGCAGTAAAGTTCTGCCGCTCCATTGGTTACCAGGGCGCGGGAACCGTGGAATTCTTGGTCGATGAAAAGGGCAACCACGTCTTCATCGAAATGAACCCACGTATCCAGGTTGAGCACACCGTGACTGAAGAAGTCACCGAGGTGGACCTGGTGAAGGCGCAGATGCGCTTGGCTGCTGGTGCAACCTTGAAGGAATTGGGTCTGACCCAAGATAAGATCAAGACCCACGGTGCAGCACTGCAGTGCCGCATCACCACGGAAGATCCAAACAACGGCTTCCGCCCAGATACCGGAACTATCACCGCGTACCGCTCACCAGGCGGAGCTGGCGTTCGTCTTGACGGTGCAGCTCAGCTCGGTGGCGAAATCACCGCACACTTTGACTCCATGCTGGTGAAAATGACCTGCCGTGGTTCCGACTTTGAAACTGCTGTTGCTCGTGCACAGCGCGCGTTGGCTGAGTTCACCGTGTCTGGTGTTGCAACCAACATTGGTTTCTTGCGTGCGTTGCTGCGGGAAGAGGACTTCACTTCCAAGCGCATCGCCACCGGATTCATTGCCGATCACCCGCACCTCCTTCAGGCTCCACCTGCTGATGATGAGCAGGGACGCATCCTGGATTACTTGGCAGATGTCACCGTGAACAAGCCTCATGGTGTGCGTCCAAAGGATGTTGCAGCTCCTATCGATAAGCTGCCTAACATCAAGGATCTGCCACTGCCACGCGGTTCCCGTGACCGCCTGAAGCAGCTTGGCCCAGCCGCGTTTGCTCGTGATCTCCGTGAGCAGGACGCACTGGCAGTTACTGATACCACCTTCCGCGATGCACACCAGTCTTTGCTTGCGACCCGAGTCCGCTCATTCGCACTGAAGCCTGCGGCAGAGGCCGTCGCAAAGCTGACTCCTGAGCTTTTGTCCGTGGAGGCCTGGGGCGGCGCGACCTACGATGTGGCGATGCGTTTCCTCTTTGAGGATCCGTGGGACAGGCTCGACGAGCTGCGCGAGGCGATGCCGAATGTAAACATTCAGATGCTGCTTCGCGGCCGCAACACCGTGGGATACACCCCGTACCCAGACTCCGTCTGCCGCGCGTTTGTTAAGGAAGCTGCCAGCTCCGGCGTGGACATCTTCCGCATCTTCGACGCGCTTAACGACGTCTCCCAGATGCGTCCAGCAATCGACGCAGTCCTGGAGACCAACACCGCGGTAGCCGAGGTGGCTATGGCTTATTCTGGTGATCTCTCTGATCCAAATGAAAAGCTCTACACCCTGGATTACTACCTAAAGATGGCAGAGGAGATCGTCAAGTCTGGCGCTCACATCTTGGCCATTAAGGATATGGCTGGTCTGCTTCGCCCAGCTGCGGTAACCAAGCTGGTCACCGCACTGCGCCGTGAATTCGATCTGCCAGTGCACGTGCACACCCACGACACTGCGGGTGGCCAGCTGGCAACCTACTTTGCTGCAGCTCAAGCTGGTGCAGATGCTGTTGACGGTGCTTCCGCACCACTGTCTGGCACCACCTCCCAGCCATCCCTGTCTGCCATTGTTGCTGCATTCGCGCACACCCGTCGCGATACCGGTTTGAGCCTCGAGGCTGTTTCTGACCTCGAGCCGTACTGGGAAGCAGTGCGCGGACTGTACCTGCCATTTGAGTCTGGAACCCCAGGCCCAACCGGTCGCGTCTACCGCCACGAAATCCCAGGCGGACAGTTGTCCAACCTGCGTGCACAGGCCACCGCACTGGGCCTTGCGGATCGTTTCGAACTCATCGAAGACAACTACGCAGCCGTTAATGAGATGCTGGGACGCCCAACCAAGGTCACCCCATCCTCCAAGGTTGTTGGCGACCTCGCACTCCACCTCGTTGGTGCGGGTGTGGATCCAGCAGACTTTGCTGCCGATCCACAAAAGTACGACATCCCAGACTCTGTCATCGCGTTCCTGCGCGGCGAGCTTGGTAACCCTCCAGGTGGCTGGCCAGAGCCACTGCGCACCCGCGCACTGGAAGGCCGCTCCGAAGGCAAGGCACCTCTGACGGAAGTTCCTGAGGAAGAGCAGGCGCACCTCGACGCTGATGATTCCAAGGAACGTCGCAATAGCCTCAACCGCCTGCTGTTCCCGAAGCCAACCGAAGAGTTCCTCGAGCACCGTCGCCGCTTCGGCAACACCTCTGCGCTGGATGATCGTGAATTCTTCTACGGCCTGGTCGAAGGCCGCGAGACTTTGATCCGCCTGCCAGATGTGCGCACCCCACTGCTTGTTCGCCTGGATGCGATCTCTGAGCCAGACGATAAGGGTATGCGCAATGTTGTGGCCAACGTCAACGGCCAGATCCGCCCAATGCGTGTGCGTGACCGCTCCGTTGAGTCTGTCACCGCAACCGCAGAAAAGGCAGATTCCTCCAACAAGGGCCATGTTGCTGCACCATTCGCTGGTGTTGTCACCGTGACTGTTGCTGAAGGTGATGAGGTCAAGGCTGGAGATGCAGTCGCAATCATCGAGGCTATGAAGATGGAAGCAACAATCACTGCTTCTGTTGACGGCAAAATCGATCGCGTTGTGGTTCCTGCTGCAACGAAGGTGGAAGGTGGCGACTTGATCGTCGTCGTTTCCTAA

*cgl2079*:

ATGACAGTTGATGAGCAGGTCTCTAACTATTACGACATGCTTCTGAAGCGCAATGCTGGCGAGCCTGAATTTCACCAGGCAGTGGCAGAGGTTTTGGAATCTTTGAAGATCGTCCTGGAAAAGGACCCTCATTACGCTGATTACGGTCTCATCCAGCGCCTGTGCGAGCCTGAGCGTCAGCTCATCTTCCGTGTGCCTTGGGTTGATGACCAGGGCCAGGTCCACGTCAACCGTGGTTTCCGCGTGCAGTTCAACTCTGCACTTGGACCATACAAGGGCGGCCTGCGCTTCCACCCATCTGTAAACCTGGGCATTGTGAAGTTCCTGGGCTTTGAGCAGATCTTTAAAAACTCCCTAACCGGCCTGCCAATCGGTGGTGGCAAGGGTGGATCCGACTTCGACCCTAAGGGCAAGTCCGATCTGGAAATCATGCGTTTCTGCCAGTCCTTCATGACCGAGCTACACCGCCACATCGGTGAGTACCGCGACGTTCCTGCAGGTGACATCGGAGTTGGTGGCCGCGAGATCGGTTACCTGTTTGGCCACTACCGTCGCATGGCTAACCAGCACGAGTCCGGCGTTTTGACCGGTAAGGGCCTGACCTGGGGTGGATCCCTGGTCCGCACCGAGGCAACTGGCTACGGCTGCGTTTACTTCGTGAGTGAAATGATCAAGGCTAAGGGCGAGAGCATCAGCGGCCAGAAGATCATCGTTTCCGGTTCCGGCAACGTAGCAACCTACGCGATTGAAAAGGCTCAGGAACTCGGCGCAACCGTTATTGGTTTCTCCGATTCCAGCGGTTGGGTTCATACCCCTAACGGCGTTGACGTGGCTAAGCTCCGCGAAATCAAGGAAGTTCGTCGCGCACGCGTATCCGTGTACGCCGACGAAGTTGAAGGCGCAACCTACCACACCGACGGTTCCATCTGGGATCTCAAGTGCGATATCGCTCTTCCTTGTGCAACTCAGAACGAGCTCAACGGCGAGAACGCTAAGACTCTTGCAGACAACGGCTGCCGTTTCGTTGCTGAAGGCGCGAACATGCCTTCCACCCCTGAGGCTGTTGAGGTCTTCCGTGAGCGCGACATCCGCTTCGGACCAGGCAAGGCAGCTAACGCTGGTGGCGTTGCAACCTCCGCTCTGGAGATGCAGCAGAACGCTTCGCGCGATTCCTGGAGCTTCGAGTACACCGACGAGCGCCTCCAGGTGATCATGAAGAACATCTTCAAGACCTGTGCAGAGACCGCAGCAGAGTATGGACACGAGAACGATTACGTTGTCGGCGCTAACATTGCTGGCTTCAAGAAGGTAGCTGACGCGATGCTGGCACAGGGCGTCATCTAA

*pckA*：

ATGTTTCACCCATGACCGATCTGAACCAACTCACTCAGGAACTCGGCGCACTGGGTATCCACGACGTGCAGGAGGTGGTGTACAACCCATCCTACGAATTACTCTTTGCAGAAGAGACCAAGCCTGGCCTGGAGGGCTATGAAAAGGGCACCGTGACTAACCAAGGCGCAGTGGCAGTGAATACCGGCATTTTCACCGGCCGCTCCCCAAAGGACAAGTACATCGTGCTGGATGACAAGACCAAGGACACCGTCTGGTGGACCTCCGAGAAGGTGAAAAACGACAATAAACCTATGTCCCAAGACACCTGGAACTCTCTGAAGGGTCTGGTGGCCGATCAACTCTCTGGCAAGCGCCTGTTTGTCGTGGACGCCTTCTGCGGCGCAAACAAAGATACTCGCCTGGCAGTGCGCGTGGTGACTGAGGTGGCCTGGCAGGCCCACTTCGTGACCAACATGTTCATCCGCCCATCCGCCGAGGAATTAAAGGGTTTCAAGCCAGACTTCGTGGTCATGAACGGCGCAAAGTGCACTAACCCAAACTGGAAGGAGCAGGGCCTGAACTCCGAAAACTTTGTGGCATTCAACATCACCGAGGGCGTGCAACTCATCGGCGGTACTTGGTACGGCGGCGAAATGAAGAAGGGTATGTTCTCCATGATGAACTATTTCCTCCCACTGCGCGGCATTGCCTCCATGCACTGCTCCGCAAACGTGGGCAAGGACGGCGATACCGCAATTTTCTTCGGTCTGTCCGGCACTGGCAAGACCACTCTGTCCACCGACCCTAAGCGCCAGCTGATCGGTGACGATGAACACGGTTGGGACGACGAGGGTGTGTTCAACTTCGAGGGCGGCTGTTACGCAAAGACTATCAACCTCTCCGCTGAAAACGAACCAGACATCTACGGCGCCATCAAGCGCGATGCCCTGCTGGAAAACGTCGTCGTGCTCGATAACGGCGATGTGGATTACGCCGACGGCTCCAAAACCGAAAACACCCGCGTCTCCTACCCAATCTATCATATCCAAAACATCGTGAAGCCAGTGTCCAAGGCAGGCCCAGCAACCAAGGTGATCTTTCTGTCCGCCGATGCATTCGGTGTGCTGCCACCAGTGTCCAAACTGACCCCAGAGCAAACTAAGTATTACTTCCTCTCTGGCTTTACCGCCAAACTGGCAGGCACCGAACGCGGCATCACCGAACCAACTCCTACCTTCTCCGCCTGCTTCGGCGCAGCATTCCTGTCTCTGCATCCAACCCAGTACGCAGAGGTGCTGGTCAAACGTATGCAAGAATCCGGCGCCGAAGCATACCTCGTGAACACCGGCTGGAACGGCACCGGTAAGCGCATCTCCATTAAGGACACCCGCGGCATCATCGATGCCATCCTGGATGGCTCCATCGATAAGGCCGAGATGGGCTCTCTGCCAATCTTCGACTTCTCCATCCCTAAAGCCCTGCCTGGCGTGAATCCAGCCATCCTCGATCCTCGCGATACCTACGCAGATAAGGCCCAGTGGGAAGAGAAGGCACAGGATCTGGCAGGCCGCTTCGTGAAGAACTTTGAGAAGTACACCGGTACTGCCGAAGGTCAGGCACTGGTGGCCGCAGGTCCAAAGGCATAA
